# Cardiomyocyte‐Specific Deletion of Sirtuin 5 Accelerates the Development of Heart Failure Upon Dysregulating Purine Metabolism

**DOI:** 10.1111/apha.70120

**Published:** 2025-10-17

**Authors:** Nikole J. Byrne, Christoph Koentges, Katharina Pfeil, Julia C. Lueg, Sayan Bakshi, Aleksandre Tarkhnishvili, Ivan Vosko, Johannes Gollmer, Laura C. Birkle, Thomas Rathner, Stephan Birkle, Sibai Tang, Clara Rau, Michael M. Hoffmann, Katja E. Odening, Stephen Barnes, Landon Shay Wilson, Senka Ljubojevic‐Holzer, Markus Wallner, Dirk von Lewinski, Peter Rainer, Simon Sedej, Harald Sourij, Christoph Bode, Adam R. Wende, Andreas Zirlik, Heiko Bugger

**Affiliations:** ^1^ Department of Cardiology, University Heart Center Graz Medical University of Graz Graz Austria; ^2^ Department of Cardiology and Angiology I Heart Center Freiburg University Freiburg Germany; ^3^ Division of Molecular and Cellular Pathology, Department of Pathology University of Alabama at Birmingham Birmingham Alabama USA; ^4^ Faculty of Medicine University of Freiburg Freiburg Germany; ^5^ Institute for Clinical Chemistry and Laboratory Medicine Medical Center—University of Freiburg Freiburg Germany; ^6^ Translational Cardiology, Department of Cardiology Bern University Hospital Bern Switzerland; ^7^ Pharmacology and Toxicology University of Alabama at Birmingham Birmingham Alabama USA; ^8^ BioTechMed Graz Graz Austria; ^9^ Institute of Physiology, Faculty of Medicine University of Maribor Maribor Slovenia; ^10^ Division of Endocrinology and Dialectology, Department of Internal Medicine Medical University of Graz Graz Austria

**Keywords:** adenosine kinase, heart failure, purine metabolism, SIRT5, sirtuin

## Abstract

**Aim:**

Sirtuin 5 (SIRT5), a mitochondrial NAD^+^‐dependent deacylase, regulates fundamental cellular pathways, including energy substrate metabolism. The current study is designed to better elucidate the role of SIRT5 in the development of heart failure (HF).

**Methods:**

Mice with cardiomyocyte‐specific deletion (*cSirt5* ^−/−^) or overexpression (c*Sirt5*‐Tg) of SIRT5 were generated and subjected to chronic pressure overload by transverse aortic constriction (TAC) or Sham surgery. Cardiac structure and function were assessed by echocardiography, isolated heart perfusions, and histology. MS‐based metabolomics and bulk RNA sequencing were used to explore metabolic and molecular signatures.

**Results:**

c*Sirt5*‐Tg mice had similar cardiac structure and function compared to control mice, whereas c*Sirt5* ^−/−^ mice displayed exacerbated cardiac dilation and dysfunction following TAC, measured both in vivo by echocardiography and ex vivo in isolated heart perfusions. Metabolomics revealed accumulation of inosine and hypoxanthine, and depletion of adenosine, adenine, AMP, and ADP in c*Sirt5* ^−/−^ hearts and following TAC, indicating dysregulation of purine metabolism. RNA‐sequencing uncovered upregulation of purine‐nucleoside phosphorylase and 5′ nucleotidase, and downregulation of adenosine kinase (ADK) in c*Sirt5* ^−/−^ hearts following TAC, indicating dysregulation at the interface of adenosine nucleotide salvage and purine degradation in the absence of SIRT5. Analyses of left ventricular tissue of patients with HF revealed reduced SIRT5 expression correlating with reduced ADK expression.

**Conclusion:**

Loss of SIRT5 in cardiomyocytes aggravates cardiac remodeling and dysfunction in response to chronic pressure overload, involving ATP precursor depletion due to transcriptional dysregulation of cardiac purine metabolism.

**Practitioner Points**

- Loss of sirtuin 5 (SIRT5) in cardiomyocytes aggravates cardiac remodeling and dysfunction following chronic pressure overload associated with depletion of adenine nucleotides and accumulation of purine metabolites.
- SIRT5 deficiency induces increased purine degradation by altering expression of enzymes of purine metabolism.
- Human failing hearts display reduced SIRT5 and adenosine kinase (ADK) expression, suggesting significance of impaired purine metabolism in heart failure pathogenesis.

**Abbreviations**
4‐HNE: 4‐hydroxynonenal
ACAA: acetyl‐CoA acyltransferase
ADA: adenosine deaminase
ADARB: ADA acting on RNA specific B
ADK: adenosine kinase
ADP: adenosine diphosphate
ADSL: adenylosuccinate lyase
AMDP: AMP deaminase
AMP: adenosine monophosphate
APRT: adenine phosphoribosyl transferase
ATIC: 5‐aminoimidazole‐4‐carboxamide ribonucleotide formyltransferase/IMP cyclohydrolase
ATP: adenosine triphosphate
BW: body weight
CANT: calcium activated nucleotidase
CAT: catalase
CCN: cellular communication network factor
CDP: cytidine diphosphate
CMP: cytidine monophosphate
CO: cardiac output
COL: collagen
c*Sirt5* ^−/−^
: cardiomyocyte‐specific deletion of *Sirt5*
CTP: cytidine triphosphate
d: diastolic
DCM: dilated cardiomyopathy
DEG: differentially expressed genes
DHP: dihydropyrimidinase
DPYD: dihydropyrimidine dehydrogenase
ECH: enoyl‐coA hydratase
EDV: end‐diastolic volume
EF: ejection fraction
ENTPD: ectonucleoside triphosphate diphosphohydrolase
ESV: end‐systolic volume
ETC: electron transport chain
FA: fatty acid
FAD: flavin adenine dinucleotide
FAO: fatty acid oxidation
FC: fold change
FS: fractional shortening
GCLM: glutamate‐cysteine ligase modifier subunit
GDP: guanosine diphosphate
GMP: guanosine monophosphate
GMPS: GMP synthetase
GNS: glucosamine (N‐acetyl)‐6‐sulfatase
GPX: glutathione peroxidase
GSH: reduced glutathione
GSR: glutathione disulfide
GSSG: glutathione disulfide
GTP: guanosine triphosphate
HADH: hydroxyacyl‐coenzyme A dehydrogenase
HF: heart failure
HPRT: hypoxanthine guanine phosphoribosyl transferase
HR: heart rate
HW: heart weight
IMP: inosine monophosphate
IMPDH: IMP dehydrogenase
LV: left ventricular
LVAW: LV anterior wall
LVID: LV internal diameter
LVPW: LV posterior wall
MVO2: myocardial oxygen consumption
N.D: not detectable
NT5E: 5′ nucleotidase
OXPHOS: oxidative phosphorylation
PNP: purine‐nucleoside phosphorylase
PPAT: phosphoribosyl pyrophosphate amidotransferase
PRDX: thioredoxin‐dependent peroxide reductase
PRPS: phosphoribosyl pyrophosphate synthetase
ROS: reactive oxygen species
RPLP0: ribosomal protein large P0
s: systolic
SIRT5: sirtuin 5
SOD: superoxide dismutase
SV: stroke volume
TAC: transverse aortic constriction
TGFB: transforming growth factor beta
TL: tibia length
TRXN: thioredoxin
UCK: uridine‐cytidine kinase
UDP: uridine diphosphate
UMP: uridine monophosphate
UMPS: UMP synthetase
UOX: urate oxidase
UPB: ureidopropionase, beta
UPP: uridine phosphorylase
UTP: uridine triphosphate
WGA: wheat germ agglutinin
XDH: xanthine dehydrogenase
XO: xanthine oxidase

## 1 Introduction

Sirtuins (SIRTs) are a protein family consisting of 7 members (SIRT1–7) which regulate cellular metabolism, inflammation, gene transcription, and DNA repair upon modulating post‐translational modifications and gene transcription [1]. Of the mammalian sirtuins, SIRT3, SIRT4, and SIRT5 have been reported to localize to mitochondria [2]. Whereas SIRT3 has primarily deacetylase activity [3], SIRT5 is shown to remove other acyl groups from lysine residues, particularly malonyl [4], succinyl [5] and glutaryl [6]. SIRT5 protein and mRNA are highly expressed in mouse and human heart compared with other tissues [4] and protein lysine succinylation occurs to the greatest extent in the heart [7]. Importantly, SIRT5 has been implicated in regulating the activity of many key proteins involved in energy metabolism, specifically in oxidative phosphorylation, TCA cycle, and fatty acid oxidation [8]. However, the role and potential underlying mechanisms of SIRT5 in regulating contractile function and energy metabolism in the heart are still a matter of debate. While global loss of SIRT5 alone is sufficient to promote the development of pathological cardiac hypertrophy [7] and worsen cardiac dysfunction following pressure overload [9], other studies reported no differences [10] or even attenuation of cardiac hypertrophy and dysfunction [11] upon pressure overload in mice lacking SIRT5. Using a gain‐of‐function model, Guo et al. [12] recently reported that mice with global SIRT5 overexpression were protected against left ventricular (LV) remodeling and functional deterioration following pressure overload. While detrimental effects on cardiac function in the absence of SIRT5 were related to impaired oxidative metabolism and ATP synthesis, protective effects were related to AMPK activation, inhibition of protein translation, or multitissue or prenatal effects.

Cardiac contraction and ion pump function are mainly maintained by hydrolysis of adenosine triphosphate (ATP) resulting from oxidative energy metabolism. Cardiac ATP is regenerated primarily (~70%) from the oxidation of fatty acids, while the remaining amount of ATP is generated by oxidation of glucose, lactate, ketone bodies, and amino acids. ATP hydrolysis results in the formation of adenosine diphosphate (ADP) and adenosine monophosphate (AMP), which may then be converted to downstream products, adenosine, inosine monophosphate (IMP), and hypoxanthine, via the purine degradation pathway [13]. Under healthy conditions, the balance of purine nucleotide synthesis, degradation, and salvage remains in balance. However, one of the hallmarks of heart failure (HF) is a decrease in myocardial ATP levels and turnover in relation to impaired ventricular function, which is believed to cause a failure to meet the metabolic demands of the failing heart [14, 15, 16]. Also, depletion of purine synthesis intermediates and accumulation of degradation products has been observed in HF [16, 17, 18]. Particularly, reductions in the total adenine nucleotide pool are shown to be tightly linked to deterioration of systolic function in the diseased heart [19], suggesting that dysregulated purine nucleotide balance may play an important role in the development of HF.

Cardiac contractility is predominantly accomplished by a fine‐tuned system consisting of the actin‐myosin network and a flexible high‐throughput system of energy metabolic pathways in cardiomyocytes. As such, cardiomyocytes are essential in the adaptation to increased energy demands, as occurs under conditions of increased afterload and in the development of HF. To further clarify the role of SIRT5 in cardiac function and energetics, we aimed to explore how alterations of SIRT5 levels only within cardiomyocytes may affect cardiac function and structure in a model of pressure overload‐induced hypertrophy and HF, and to use unbiased metabolomic and transcriptomic analysis to explore underlying mechanisms.

## 2 Materials and Methods

### 2.1 Animals and Treatments

This study conforms to the *Guide for the Care and Use of Laboratory Animals* published by the US National Institutes of Health and was performed after securing appropriate institutional approval by the Regierungspräsidium Freiburg (G‐14/76 and G‐18/62). All mice were housed with 12 h daylight and night cycles at 22°C in individually ventilated cages in a specific pathogen‐free facility. Laboratory standard chow and water ad libitum were fed to all animals. Transgenic *Sirt5* knockout (*Sirt5* ^−/−^) mice, on a C57BL6J background, were kindly provided by Johan Auwerx from the École Polytechnique Fédérale de Lausanne, Switzerland [7]. David Lombard from the University of Michigan, Ann Arbor, Michigan, USA kindly provided transgenic *Sirt5* overexpressing (*Sirt5*‐Tg) mice on mixed C57BL6J and 129Sv1 background. Transgenic *Sirt5* ^−/−^ and *Sirt5*‐Tg mice were crossed with hemizygote cardiomyocyte‐specific recombinase‐expressing mice (*Myh6‐cre*, Stock #011038, Jackson Laboratories) to generate cardiomyocyte‐specific *Sirt5* knockout (*cSirt5* ^*−/−*^) and cardiomyocyte‐specific *Sirt5* overexpressing (c*Sirt5*‐Tg) mice, respectively. Male mice were used in the study. Recombinase non‐expressing transgenic mice were used as controls.

### 2.2 Metabolomics

Frozen hearts were dispersed with a bead homogenizer and the methanol (ice‐cold) content adjusted to 80%. The homogenate was subjected to Bligh‐Dyer extraction and the upper aqueous phase was transferred to a new tube. The methanol content was raised to 80%. Precipitated proteins were removed by centrifugation. Blank samples without tissue were processed in a similar manner. Supernatants were evaporated to dryness under N_2_. Dried residues were reconstituted in 0.1% formic acid (200 μL). Aliquots (10 μL) were injected onto a 10 cm x 2.1 mm ID, 3 μ resin Luna Omega Polar C18 (Phenomenex, Torrance, CA, USA) column (equilibrated with 0.1% formic acid). Metabolites were eluted with a 6‐min, linear 2%–98% acetonitrile gradient in 0.1% formic acid at a flow rate of 0.5 mL/min. Eluates were passed through the electrospray ionization interface of a SCIEX (Concord, Ontario, Canada) TripleTOF 5600 mass spectrometer and negative and positive ion MS and MSMS data were recorded. Collected MS and MS/MS data were processed with MS‐DIAL (v. 4:90) against positive ion and negative ion metabolite and lipid public databases. Data were cleaned to remove contaminant ions using blank samples to create .csv files. The .csv files from the negative ion and positive ion analyses were combined for submission to MetaboAnalyst v. 5.0 (https://www.metaboanalyst.com) for univariate, multivariate, and hierarchical clustering.

### 2.3 RNA Sequencing Analysis

Following the manufacturer's instructions, whole heart RNA was isolated using the RNeasy Tissue Mini Kit (Qiagen). Isolated RNA was examined to have an RNA integrity number > 7 to ensure RNA quality. Next‐generation RNA sequencing was performed using Illumina HiSeq2000 for paired‐end 75 bp sequencing at the Heflin Genomics Core at the University of Alabama at Birmingham, USA. *Trim Galore* (0.6.6), a wrapper tool around *CutAdapt* (2.6), was used to trim adapters and low‐quality (Phred score < 20) sequences. Trimmed reads were aligned to the mouse genome (GRCm39) via *STAR* (2.7.3a). R software (version 4.0.3, R Foundation for Statistical Computing, Vienna, Austria) was used to perform downstream analysis and data visualization.

Differential gene expression was performed using *DESeq2* (1.30.1)‐based [20] negative binomial generalized linear model. First, only rows with at least 10 total reads were kept from the pre‐filtered count data. Size factor or the normalizing factor estimation for different read depths of each gene was performed with *DESeq2*. It also estimates gene‐wise dispersion via maximum likelihood, followed by adjustment with the empirical Bayes method. In the end, *DESeq2* gives normalized count data for each contrast (changes in c*Sirt5* ^−/−^ mice compared to control in Sham and TAC) by negative binomial generalized linear model fitting. The Wald test, which yields normalized read counts together with log_2_ fold changes for each contrast, *p*‐values, and adjusted *p*‐values corrected for multiple testing using the Benjamini–Hochberg method, was used to quantify and assess the significance of differential gene expression.

Heatmap visualization and hierarchical clustering were performed with Ward's minimum squared variance algorithm, and dendrograms were generated by Euclidean distance via *pheatmap* (1.0.12) using regularized logarithm transformed read counts. *EnrichR* (3.0) R interface package was employed to perform pathway enrichment analysis against databases such as Gene Ontology Biological Process 2018, Reactome 2016, Bioplanet 2019, and WikiPathways 2019 [21]. Venn diagram analyses were accomplished using *Vennplex* [22]. Transcripts from each Venn diagram group were converted into putative protein–protein interaction (PPI) networks using *Cytoscape* (v3.10.1) [23]. The Molecular COmplex DEtection (MCODE; v2.0.3) [24] plugin within *Cytoscape* was employed to cluster the PPI networks into top clusters that satisfy the criteria of clustering cutoff > 3. RNA‐seq data have been deposited to the GEO database under the accession number GSE280863.

### 2.4 Human Gene Expression Data

Procedures involving human samples were approved by the Ethical Committee of the Medical University of Graz (no. 28‐508 ex 15/16) and performed in accordance with principles outlined in the Declaration of Helsinki. Informed written consent was not feasible to obtain because the patients were not able to give the informed consent as a result of their underlying medical condition. The requirement for informed consent was thereby waived by the ethical committee. Donor hearts were explanted post‐mortem. Upon ice‐cold cardioplegia, cardiac biopsies were harvested from the left ventricular free wall, flash frozen in liquid nitrogen and stored at −80°C for further analysis. Twenty‐two patients with normal EF and no history of coronary artery disease (referred to as Non‐failing) and from 14 patients with ischemic or non‐ischemic DCM and severely reduced EF (Failing).

### 2.5 Statistical Analysis

Data are presented as Mean ± SEM using GraphPad Prism software. *N* number refers to biological replicates. Two‐group comparisons were tested by *F* test to compare variances. Datasets which were significantly different (*p* < 0.05) were then subjected to Welch's correction, where equal SD was not assumed; datasets where *F* test was not significantly different were subjected to unpaired *t* test. Four‐group comparisons were tested by an ordinary two‐way analysis of variance (ANOVA), where: §, effect of TAC; #, effect of c*Sirt5* ^−/−^; % effect of interaction. Fisher's LSD test was used for multiple comparisons. A *p* < 0.05 was considered significant.

All other Materials and Methods can be found in the Data S1. The data that support the findings of this study are available from the corresponding author upon reasonable request.

## 3 Results

### 3.1 Cardiomyocyte‐Specific Deletion of SIRT5 Aggravates Cardiac Dysfunction and Remodeling Following Pressure Overload

**FIGURE 1:**
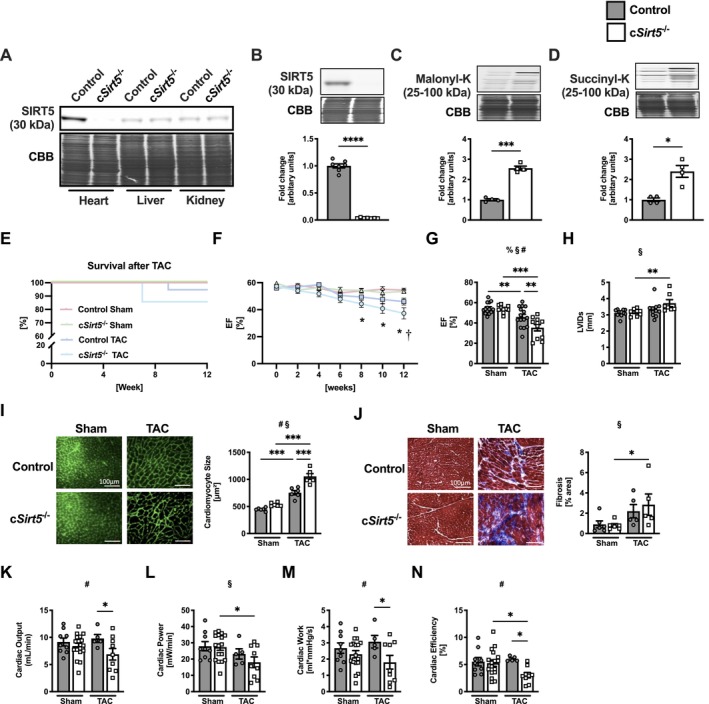
Aggravation of cardiac remodeling and dysfunction in c*Sirt5* ^−/−^ mice following TAC. (A) SIRT5 protein expression in heart, liver and kidney, and quantification of cardiac (B) SIRT5 protein (*n* = 7), (C) malonylation and (D) succinylation in control and c*Sirt5* ^−/−^ mice (*n* = 4). (E) Survival, (F) biweekly EF, (G) final EF, (H) LVIDs (*n* = 10–15), (I) cardiomyocyte size and (J) myocardial fibrosis of control and c*Sirt5* ^−/−^ mice at 12 weeks following Sham or TAC surgery (*n* = 6). Ex vivo (K) cardiac output, (L) cardiac power, (M) cardiac work, and (N) cardiac efficiency measured in isolated working hearts of control and c*Sirt5* ^−/−^ mice following 12 weeks Sham or TAC surgery (*n* = 5–17). c*Sirt5* ^−/−^, cardiomyocyte‐specific deletion of *Sirt5*; EF, ejection fraction; LVIDs, left‐ventricular internal diameter during systole; SIRT5, sirtuin 5; TAC, transverse aortic constriction. 2‐Way ANOVA: §, effect of TAC; #, effect of c*Sirt5* ^−/−^; % effect of interaction. *p < 0.05, **p < 0.01, ***p < 0.001 using Welch's *t* test (B–D) or Fisher's LSD test (G–N). *p < 0.05 versus respective Sham, †*p* < 0.05 versus control TAC using Fisher's LSD test (F).

We generated mice with cardiomyocyte‐specific deletion of *Sirt5* (c*Sirt5* ^−/−^) (Figure 1A,B), which display enhanced levels of cardiac malonylation (Figure 1C) and succinylation (Figure 1D) compared to control mice, and subjected them to TAC surgery to induce HF [25]. Following TAC, c*Sirt5* ^−/−^ mice displayed increased mortality (Figure 1E) and a further reduction in ejection fraction (EF) (Figure 1F,G) compared to control mice. c*Sirt5* ^−/−^ mice also had increased LV internal diameter (LVID) (Figure 1H) and end‐systolic volume (ESV) (Table 1) following TAC, which were less pronounced or absent in control mice following TAC. LV wall thickness (LVPWs) was increased in control mice following TAC, but not in c*Sirt5* ^−/−^ mice (Table 1), and heart weight (HW) was similarly increased in both c*Sirt5* ^−/−^ and control mice following TAC (Table S1). The TAC‐induced increase in cardiomyocyte cell size was significantly larger in hearts from c*Sirt5* ^−/−^ mice than in control mice (Figure 1I). Histological analysis showed that myocardial collagen content was significantly increased in c*Sirt5* ^−/−^ mice following TAC, but was not significantly different compared to TAC control mice (Figure 1J). To further confirm aggravation of cardiac dysfunction in c*Sirt5* ^−/−^ mice following TAC in vivo, cardiac function was also evaluated ex vivo in the absence of neurohumoral effects under standardized conditions of pre‐ and afterload. In line with our findings in vivo, ex vivo working heart perfusions found that cardiac output (Figure 1K), cardiac power (Figure 1L), cardiac work (Figure 1M), and cardiac efficiency (Figure 1N) were worse in c*Sirt5* ^−/−^ hearts following TAC. Collectively, these data show that cardiomyocyte‐specific deletion of SIRT5 aggravates cardiac dysfunction and promotes dilative remodeling in response to chronic pressure overload.

**TABLE 1:** Echocardiographic parameters of control and c*Sirt5* ^−/−^ mice 12 weeks following Sham or TAC surgery.

	Control Sham	c*Sirt5* ^−/−^ Sham	Control TAC	c*Sirt5* ^−/−^ TAC	2‐Way ANOVA
LVAWd (mm)	0.71 ± 0.02	0.72 ± 0.04	0.92 ± 0.04*	0.86 ± 0.06*	§
LVAWs (mm)	0.95 ± 0.03	0.94 ± 0.05	1.16 ± 0.04*	1.01 ± 0.08^†^	§
LVIDd (mm)	4.11 ± 0.05	4.17 ± 0.07	4.40 ± 0.15	4.56 ± 0.16*	§
LVIDs (mm)	3.12 ± 0.06	3.17 ± 0.08	3.54 ± 0.15*	3.92 ± 0.20* ^,^ ^†^	§
LVPWd (mm)	0.93 ± 0.05	0.84 ± 0.04	1.26 ± 0.12*	1.02 ± 0.05^†^	§
LVPWs (mm)	1.11 ± 0.05	1.09 ± 0.03	1.47 ± 0.11*	1.14 ± 0.06^†^	§ #
EF (%)	54.5 ± 1.7	53.8 ± 1.1	46.0 ± 2.6*	35.3 ± 3.1* ^,^ ^†^	% § #
FS (%)	24.2 ± 1	23.7 ± 0.7	19.7 ± 1.3*	14.4 ± 1.4* ^,^ ^†^	% § #
HR (min^−1^)	435 ± 11	459 ± 14	480 ± 13*	476 ± 13.8	§
EDV (μL)	68.3 ± 3.5	73.9 ± 3.8	89.7 ± 9.1*	98.6 ± 11.2*	§
ESV (μL)	29.8 ± 2.1	32.4 ± 1.9	48.0 ± 6.3*	65.5 ± 10.7* ^,^ ^†^	§
SV (μL)	38.5 ± 2.1	41.4 ± 2.5	41.7 ± 3.7	33.1 ± 1.4^†^	%
CO (μL/min)	16 894 ± 1028	18 914 ± 1059	19 798 ± 1765	15 873 ± 1061^†^	%

*Note:* Mean ± SEM; *n* = 14 Control Sham; *n* = 10 c*Sirt5* ^−/−^ Sham; *n* = 15 Control TAC; *n* = 11 c*Sirt5* ^−/−^ TAC; 2‐Way ANOVA: §, effect of TAC; #, effect of c*Sirt5* ^−/−^; % effect of interaction.

Abbreviations: CO, cardiac output; d, diastolic; EDV, end‐diastolic volume; EF, ejection fraction; ESV, end‐systolic volume; FS, fractional shortening; HR, heart rate; LV, left ventricular; LVAW, LV anterior wall; LVID, LV internal diameter; LVPW, LV posterior wall; s, systolic; SIRT5, sirtuin 5; SV, stroke volume; TAC, transverse aortic constriction.

* *p* < 0.05 versus respective Sham.

^†^ *p* < 0.05 versus control TAC using Fisher's LSD test.

### 3.2 Cardiomyocyte‐Specific Overexpression of SIRT5 Has No Effect on Cardiac Dysfunction and Remodeling Following Pressure Overload

**FIGURE 2:**
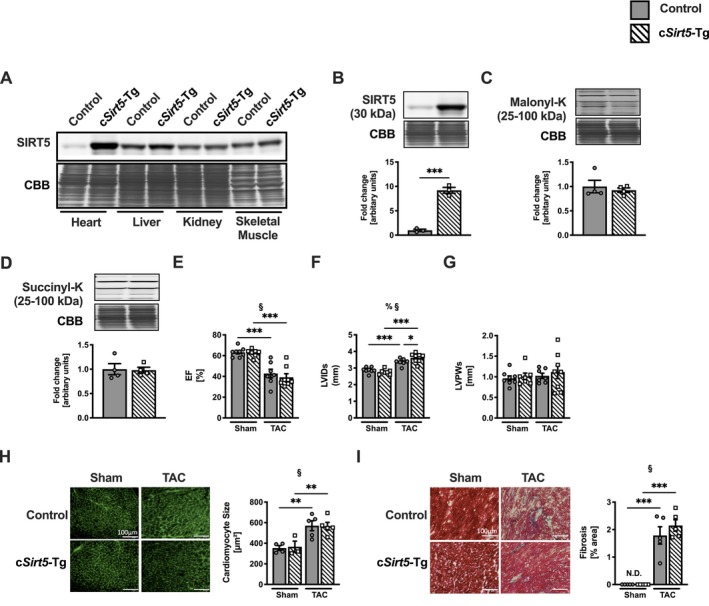
No effects on cardiac structure and function in c*Sirt5*‐Tg mice following TAC. (A) SIRT5 protein expression in heart, liver, kidney, and skeletal muscle and cardiac (B) SIRT5 protein, (C) malonylation and (D) succinylation in hearts of control and c*Sirt5*‐Tg mice (*n* = 4). (E) EF, (F) LVIDs, (G) LVPWs (*n* = 7–8), (H) cardiomyocyte size and (I) myocardial fibrosis (*n* = 4–6) of control and c*Sirt5*‐Tg mice at 8 weeks following Sham or TAC surgery. c*Sirt5*‐Tg, cardiomyocyte‐specific overexpression of *Sirt5*; EF, ejection fraction; ESV, end‐systolic volume; LVIDs, left‐ventricular internal diameter during systole; N.D., not detectable; SIRT5, sirtuin 5; TAC, transverse aortic constriction. 2‐Way ANOVA: §, effect of TAC; % effect of interaction. *p < 0.05, **p < 0.01, ***p < 0.001 using Welch's (B) or unpaired (C, D) *t* test or Fisher's LSD test (E–I).

Next, we generated mice with cardiomyocyte‐specific overexpression of *Sirt5* (c*Sirt5*‐Tg), displaying a 10‐fold increase in cardiac SIRT5 protein expression compared to control mice (Figure 2A,B). Interestingly, we observed no difference in lysine succinylation (Figure 2C) and malonylation (Figure 2D) in c*Sirt5*‐Tg compared to control hearts. We observed a similar decline in EF between c*Sirt5*‐Tg and control mice following TAC (Figure 2E). While LVID (Figure 2F) and ESV (Table 2) were mildly increased in c*Sirt5*‐Tg compared to control mice following TAC, heart weight (Table S2), LV wall thickness (Figure 2G), cardiomyocyte cell size (Figure 2H), and the degree of myocardial fibrosis (Figure 2I) were similar in c*Sirt5*‐Tg and control mice following TAC. Thus, cardiomyocyte‐specific overexpression of SIRT5 did not affect cardiac dysfunction and remodeling in response to chronic pressure overload. Based on this, we chose to focus all subsequent experiments on c*Sirt5* ^−/−^ mice, which showed a clear cardiac impairment.

**TABLE 2:** Echocardiographic parameters of control and c*Sirt5*‐Tg mice 8 weeks following Sham or TAC surgery.

	Control Sham	c*Sirt5‐*Tg Sham	Control TAC	c*Sirt5‐*Tg TAC	2‐Way ANOVA
LVAWd (mm)	0.66 ± 0.03	0.72 ± 0.04	0.94 ± 0.05*	0.84 ± 0.04*	§
LVAWs (mm)	1.06 ± 0.03	1.07 ± 0.07	1.18 ± 0.07	1.09 ± 0.04	
LVIDd (mm)	4.09 ± 0.06	3.91 ± 0.09	4.12 ± 0.07	4.33 ± 0.07* ^,^ ^†^	% §
LVIDs (mm)	2.88 ± 0.06	2.77 ± 0.06	3.37 ± 0.08*	3.62 ± 0.10* ^,^ ^†^	% §
LVPWd (mm)	0.96 ± 0.06	1.01 ± 0.08	1.02 ± 0.06	1.12 ± 0.16	
LVPWs (mm)	1.29 ± 0.06	1.32 ± 0.09	1.25 ± 0.07	1.32 ± 0.17	
EF (%)	63.3 ± 1.6	62.7 ± 1.4	42.8 ± 4.2*	39.2 ± 3.4*	§
FS (%)	29.5 ± 1.1	29.1 ± 1	18.1 ± 2.1*	16.3 ± 1.8*	§
HR (min^−1^)	434 ± 16	425 ± 38	469 ± 25	467 ± 11	
EDV (μL)	68.6 ± 2.9	60.4 ± 4.6	70.4 ± 3.6	81.6 ± 3.7* ^,^ ^†^	% §
ESV (μL)	24.2 ± 1.4	21.5 ± 1.4	38.7 ± 2.5*	48.4 ± 3.7* ^,^ ^†^	% §
SV (μL)	44.5 ± 2.2	38.9 ± 3.4	31.6 ± 3.8*	33.2 ± 2.9	§
CO (μL/min)	19 315 ± 1206	16 963 ± 2598	14 977 ± 2216	15 674 ± 1643	

*Note:* Mean ± SEM; *n* = 8 Control Sham; *n* = 7 cSirt5‐Tg Sham; *n* = 7 Control TAC; *n* = 8 cSirt5‐Tg TAC; 2‐Way ANOVA: §, effect of TAC; #, effect of cSirt5‐Tg; %, effect of interaction.

Abbreviations: CO, cardiac output; d, diastolic; EDV, end‐diastolic volume; EF, ejection fraction; ESV, end‐systolic volume; FS, fractional shortening; HR, heart rate; LV, left ventricular; LVAW, LV anterior wall; LVID, LV internal diameter; LVPW, LV posterior wall; s, systolic; SIRT5, sirtuin 5; SV, stroke volume; TAC, transverse aortic constriction.

* *p* < 0.05 versus respective Sham.

^†^ *p* < 0.05 versus control TAC using Fisher's LSD test.

### 3.3 Cardiomyocyte‐Specific Overexpression of SIRT5 Does Not Increase Myocardial Lipid Peroxidation Following Pressure Overload

Since loss of SIRT5 aggravated cardiac dysfunction and remodeling (Figure 1), and knowing that SIRT5 has been previously shown to protect against ROS‐induced damage [26], we explored a role for oxidative stress by evaluation of lipid peroxidation. Total myocardial (Figure S1A) and mitochondrial (Figure S1B) levels of 4‐hydroxynonenal (4‐HNE), an indicator of lipid peroxidation, were similar between groups, whereas TBARS assay demonstrated a decrease of malondialdehyde (MDA) in c*Sirt5* ^−/−^ hearts following TAC (Figure S1C). While gene expression of superoxide dismutase 2 (*Sod2*) and catalase (*Cat*) was not different between groups, gene expression of thioredoxin 1 (*Trxn1*), peroxidase 3 (*Prdx3*), glutathione peroxidase 1 and 4 (*Gpx1*, *Gpx4*) were markedly elevated in c*Sirt5* ^−/−^ compared to control hearts following TAC (Figure S1D). Thus, while increased expression of antioxidant genes suggests increased detoxification of ROS, the absence of increased lipid peroxidation might explain, at least in part, that cardiac ROS homeostasis was preserved in c*Sirt5* ^−/−^ mice following TAC and, therefore, is unlikely to be responsible for exacerbation of cardiac dysfunction in the absence of SIRT5 following pressure overload.

### 3.4 Cardiomyocyte‐Specific Deletion of SIRT5 Dampens Changes in Myocardial Gene Expression Following Pressure Overload

**FIGURE 3:**
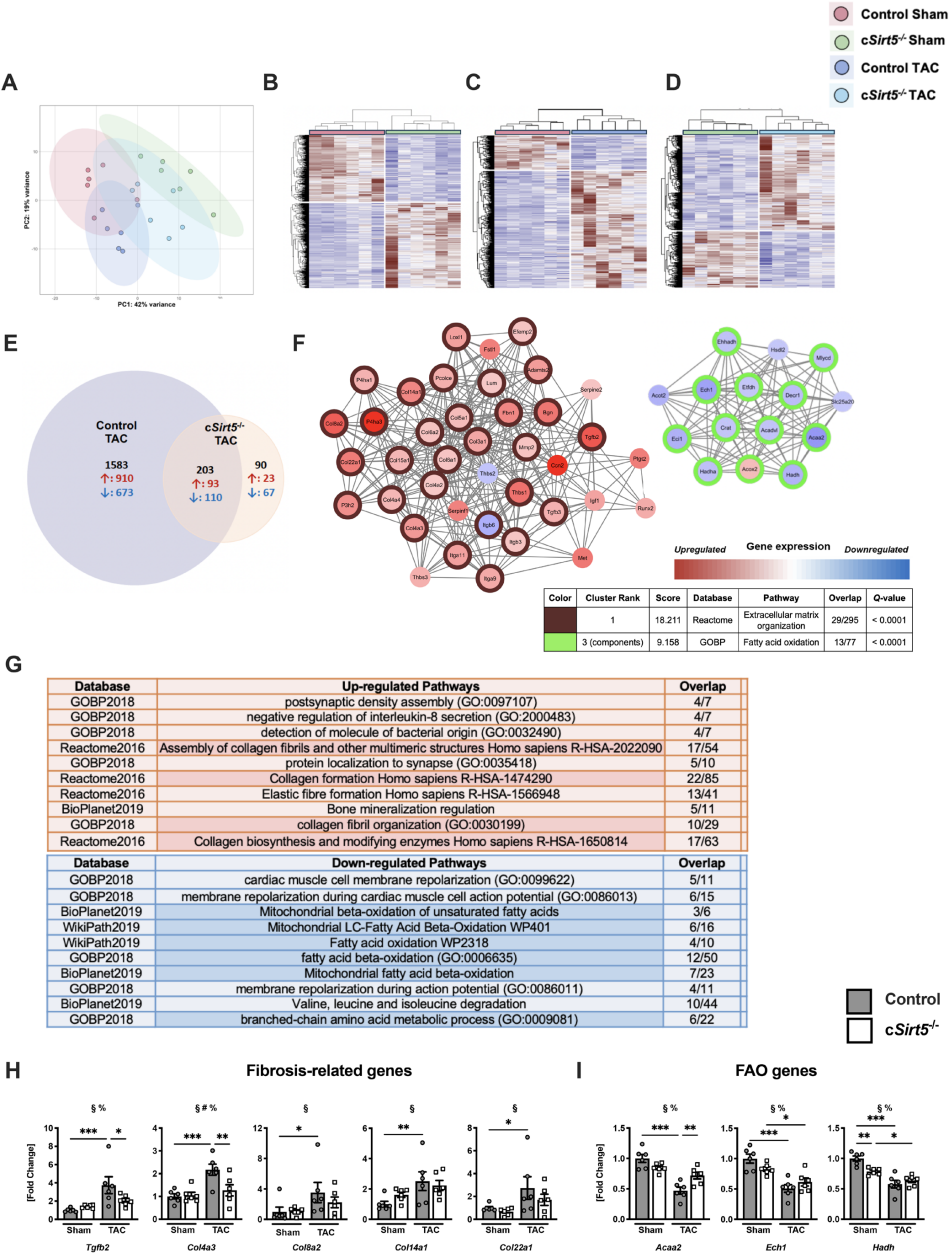
Transcriptional reprogramming of c*Sirt5* ^−/−^ hearts following TAC. (A) PCA plot, and heatmap of DEGs in (B) control Sham versus TAC, (C) control Sham versus c*Sirt5* ^−/−^ Sham, and (D) control TAC versus c*Sirt5* ^−/−^ TAC, (E) Venn diagrams of overlapping DEGs, (F) DEG clusters and (G) pathway analysis of DEGs by GOBP2018, Reactome 2016, BioPlanet2019 and WikiPath2019 in hearts of c*Sirt5* ^−/−^ compared to control mice 12 weeks following Sham or TAC surgery. Myocardial expression of genes related to (H) fibrosis and (I) FAO in control and *cSirt5* ^*−/−*^ mice 12 weeks following Sham or TAC surgery (*n* = 6). DEGs determined based on a Wald test (*p* < 0.1 and FC > 1.5) with Benjamini–Hochberg post hoc adjustment. ACAA, acetyl‐coA acyltransferas; CAT, catalase; CCN, cellular communication network factor; COL, collagen; c*Sirt5* ^−/−^, cardiomyocyte‐specific deletion of *Sirt5*; DEG, differentially expressed genes; ECH, enoyl‐coA hydratase; FAO, fatty acid oxidation; FC, fold change; HADH, hydroxyacyl‐coenzyme A dehydrogenase; SIRT5, sirtuin 5; TAC, transverse aortic constriction; TGFB, transforming growth factor beta. 2‐Way ANOVA: §, effect of TAC; #, effect of c*Sirt5* ^−/−^; % effect of interaction. *p < 0.05, **p < 0.01, ***p < 0.001 using Fisher's LSD test (H, I).

**FIGURE 4:**
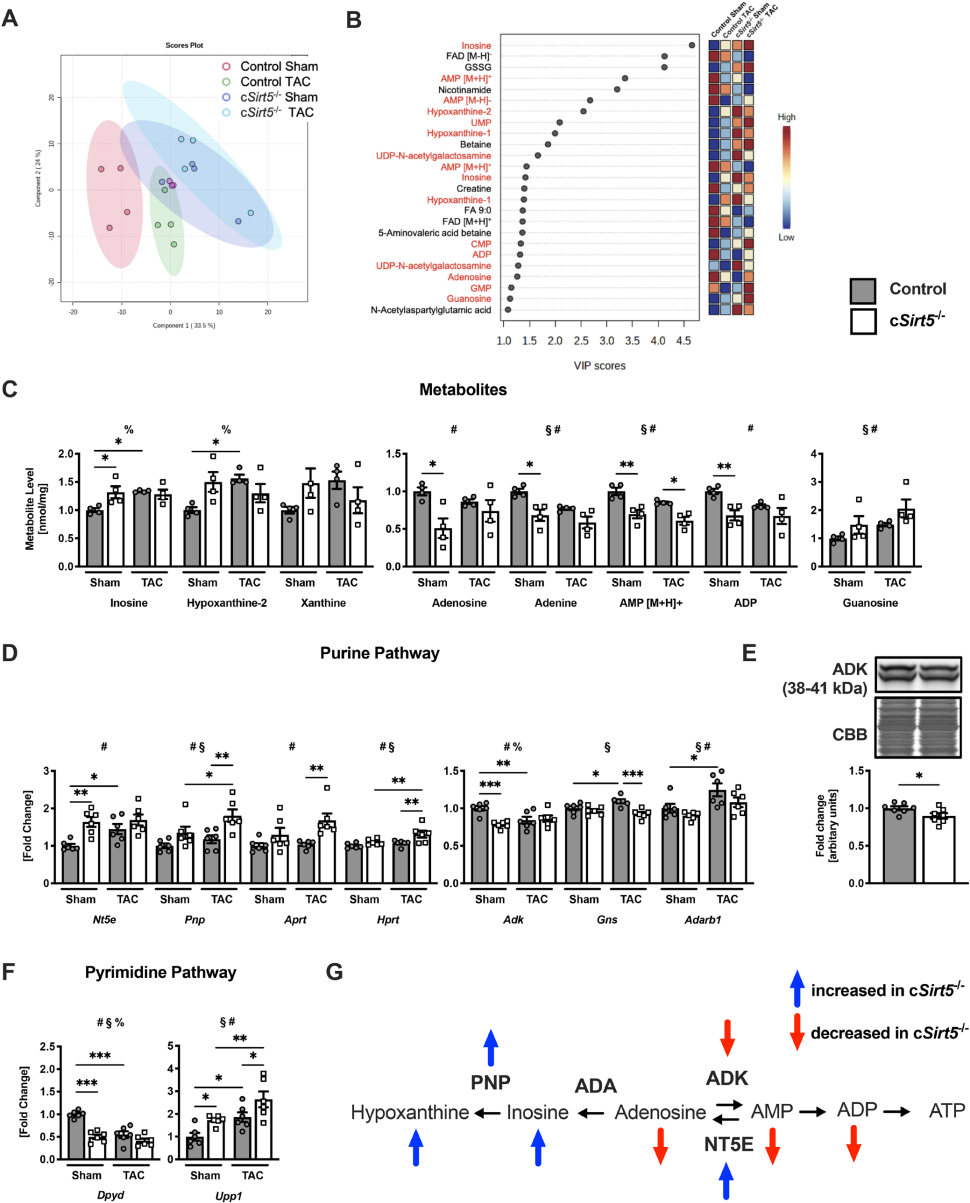
Altered purine and pyrimidine metabolism in c*Sirt5* ^−/−^ hearts following TAC. (A) PLS‐DA plots and (B) VIP scores of top 25 metabolites in c*Sirt5* ^−/−^ hearts compared to control 12 weeks following Sham or TAC surgery (*n* = 6). Changes in myocardial (C) metabolite level and expression of (D) genes related to purine pathway, (E) ADK protein, and (F) genes related to pyrimidine pathway in control and c*Sirt5* ^−/−^ hearts (*n* = 6). (G) Schematic illustrating alterations in gene expression and metabolites involved in purine pathway conversion of adenosine to ATP via ADK or to hypoxanthine via ADA and PNP in c*Sirt5* ^−/−^ hearts. VIP scores ≥ 1.0 were considered significant. ADA, adenosine deaminase; ADARB, ADA acting on RNA specific B; ADK, adenosine kinase; ADP, adenosine diphosphate; AMP, adenosine monophosphate; APRT, adenine phosphoribosyl transferase; ATP, adenosine triphosphate; DPYD, dihydropyrimidine dehydrogenase; GNS, glucosamine (N‐acetyl)‐6‐sulfatase; HPRT, hypoxanthine guanine phosphoribosyl transferase; NT5E, 5′ nucleotidase, ecto; PLS‐DA, partial least squares‐discriminant analysis; PNP, purine‐nucleoside phosphorylase; PRPS, phosphoribosyl pyrophosphate synthetase; SIRT5, sirtuin 5; TAC, transverse aortic constriction; UPP, uridine phosphorylase. 2‐Way ANOVA: §, effect of TAC; #, effect of c*Sirt5*‐Tg; % effect of interaction. *p < 0.05, **p < 0.01, ***p < 0.001 using unpaired *t* test (E) or Fisher's LSD test (C, D, F).

To further explore mechanisms underlying aggravation of cardiac dysfunction and remodeling in c*Sirt5* ^−/−^ mice following TAC, we performed an unbiased transcriptomic profiling using bulk RNA sequencing. We defined differentially regulated genes (DEGs) as those whose expression levels were above or below a 1.5‐fold change compared to controls, and with an adjusted *p‐*value (*Q*‐value) of < 0.1. Principal component analysis (PCA) (Figure 3A) and heatmaps showed that the transcriptomic profiles were markedly different in control hearts following TAC (Figure 3B) and in c*Sirt5* ^−/−^ hearts following Sham (Figure 3C) or TAC (Figure 3D) surgery compared to control hearts. Specifically, the analysis revealed a total of 1786 DEGs in control hearts following TAC, whereas only 293 DEGs were detected in c*Sirt5* ^−/−^ hearts following TAC (Figure 3E). These findings suggest that c*Sirt5* ^−/−^ hearts have an impaired response to several TAC‐induced changes in gene expression. The pathway enrichment analysis conducted on the transcripts uniquely altered in control hearts following TAC revealed that the top PPI cluster (Figure 3F) and 4 of the top 10 pathways identified with only upregulated DEGs were related to collagen fiber formation and extracellular matrix remodeling (Figure 3G). However, based on the fact that both the expression of fibrosis‐related genes (Figure 3H) and collagen content (Figure 1J) were not further increased in c*Sirt5* ^−/−^ compared to control hearts following TAC, it suggests that the exacerbated response of hearts lacking SIRT5 to pressure overload may not be attributed to worsened fibrotic remodeling. Moreover, components of the top 3 PPI clusters (Figure 3F) and 6 of the top 10 pathways with significant enrichment of downregulated DEGs induced in control hearts following TAC were related to mitochondrial FAO and branched‐chain amino acid metabolism (Figure 3G). In contrast, suppression of FAO following TAC was absent in c*Sirt5* ^−/−^ hearts, suggesting that SIRT5 may be required for metabolic reprogramming in response to pressure overload, thereby contributing to the worsening of cardiac function.

### 3.5 Cardiomyocyte‐Specific Deletion of SIRT5 Dysregulates Myocardial Purine Metabolism Following Pressure Overload

To expand our analysis of metabolic mechanisms potentially contributing to exacerbated cardiac dysfunction in c*Sirt5* ^−/−^ hearts, we performed unbiased MS/MS‐based metabolomics. Partial least squares‐discriminant analysis (PLS‐DA) plots showed clear separation for metabolite patterns between Sham and TAC in both control and c*Sirt5* ^−/−^ hearts and between control and c*Sirt5* ^−/−^ Sham hearts. However, metabolite levels in c*Sirt5* ^−/−^ Sham hearts overlapped with all other groups (Figure 4A). A total of 166 metabolites were annotated, of which variables with a VIP score of ≥ 1 were considered relevant. Inosine, an intermediate in the degradation of purines, showed the highest VIP score (Figure 4B). Moreover, 16 of the top 20 metabolites were noted to be involved in the purine and pyrimidine metabolism pathway (indicated in red) (Figure 4B). Inosine and its degradation product, hypoxanthine‐2, increased in the hearts of c*Sirt5* ^−/−^ Sham mice but were also significantly increased in control hearts following TAC (Figure 4C). In contrast, adenosine, adenine, AMP, and ADP were significantly reduced in the hearts of c*Sirt5* ^−/−^ Sham mice (Figure 4C). Thus, our metabolite data highlight a dysregulation of purine metabolism with a shift away from the salvage of adenine nucleotides towards increased degradation of purine metabolites in hearts of c*Sirt5* ^−/−^ mice.

To identify potential mechanisms that may involve dysregulated purine metabolism in c*Sirt5* ^−/−^ hearts, we explored related gene expression in our RNA sequencing database (Figure S2). We observed differential expression of various genes involved in purine metabolism (*Nt5e, Pnp, Aprt, Hprt, Adk, Gns*) and pyrimidine metabolism (*Dpyd, Upp1*) in c*Sirt5* ^−/−^ hearts following Sham or TAC surgery (Figure 4D,F). Of these, an increased expression of 5′ nucleotidase, ecto (*Nt5e*) and purine‐nucleoside phosphorylase (*Pnp*) may mediate increased purine degradation, whereas decreased expression of adenosine kinase (*Adk*) may result in impaired salvage of adenosine nucleotides (Figure 4D,F). In line with this, we also found significantly decreased ADK protein expression in c*Sirt5* ^−/−^ hearts (Figure 4E). Collectively, metabolite and gene expression data may suggest that SIRT5 deficiency induces a transcriptional dysregulation of enzymes at the interface of purine degradation and adenosine nucleotide salvage, thereby impairing ATP regeneration and ultimately cardiac energetics (Figure 4G).

### 3.6 Decreased SIRT5 and ADK Expression in Human Failing Hearts

**FIGURE 5:**
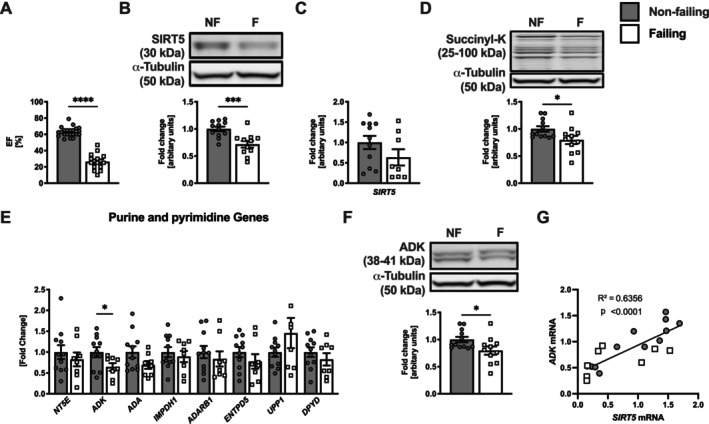
Decreased expression of SIRT5 and ADK in human failing hearts. (A) EF (*n* = 15–20), (B) SIRT5 protein, (C) *SIRT5* gene expression, and (D) total succinylation in LV heart tissue of patients with HF (*n* = 8–12). Expression of (E) purine and pyrimidine genes, (F) ADK protein, and (G) correlation of *ADK* and *SIRT5* gene expression in LV heart tissue of patients with HF (*n* = 10–12). ADA, adenosine deaminase; ADARB, ADA acting on RNA specific B; ADK, adenosine kinase; DPYD, dihydropyrimidine dehydrogenase; ENTPD, ectonucleoside triphosphate diphosphohydrolase; EF, ejection fraction; HF, heart failure; IMPDH, inosine monophosphate dehydrogenase; LV, left ventricular; NT5E, 5′ nucleotidase, ecto; SIRT5, sirtuin 5; TAC, transverse aortic constriction; UPP, uridine phosphorylase. *p < 0.05, ***p < 0.001, ****p < 0.0001 using Welch's (A) or unpaired (B–E, G) *t* test.

To explore the translational significance of the role of loss of SIRT5 in the development of HF, we assessed SIRT5 expression in LV heart tissue of patients with HF, where EF was significantly reduced (26% ± 10%), compared to healthy, age‐ and sex‐matched non‐failing control patients (63% ± 6%) (Figure 5A; Table S5). We observed a 29% and 36% reduction of SIRT5 protein expression (Figure 5B) and *SIRT5* mRNA expression (Figure 5C), respectively, in failing hearts compared to non‐failing hearts, where protein expression reached statistical significance. Total heart succinylation was not altered between groups (Figure 5D). We also assessed the expression of several genes related to purine and pyrimidine metabolism. Importantly, we observed a significant decrease in ADK gene (Figure 5E) and protein (Figure 5F) expression in human failing hearts. Of note, decreased *ADK* expression significantly correlated with the decline in *SIRT5* expression in failing and non‐failing hearts (Figure 5G). Collectively, these findings corroborate results in mice and suggest that decreased levels of SIRT5 in failing human hearts are associated with downregulation of ADK, which may aggravate myocardial energy depletion and thereby promote disease progression in HF.

## 4 Discussion

Based on controversial studies on the role of SIRT5 in cardiac physiology, we characterized mice with cardiomyocyte‐selective deletion or overexpression of SIRT5. Using both in vivo and ex vivo methodologies, we here show that cardiomyocyte‐selective deletion of SIRT5 aggravates cardiac dysfunction in response to chronic pressure overload, clearly indicating that cardiomyocyte SIRT5 is required for adaptation to increased energy demands. Integrating the results of previous studies now allows a new interpretation of the impact of SIRT5 on cardiac physiology. Our data are in line with studies demonstrating age‐dependent cardiac hypertrophy and dysfunction in mice with germline global knockout of SIRT5 [7], and a study by Hershberger et al. reporting aggravation of pathological hypertrophy and contractile dysfunction also investigated in global SIRT5 knockout mice [9]. These studies have in common that SIRT5 deletion occurred during prenatal development. However, postnatal induction of cardiomyocyte‐restricted SIRT5 deletion with onset of SIRT5 deletion no earlier than 3 weeks after birth and onset of significantly increased protein succinylation at an adult age did not affect cardiac function and structure [10], strongly suggesting that the temporal difference in SIRT5 deletion among transgenic models is critical for the cardiac phenotype. Indeed, structural development and energy metabolism of cardiomyocyte mitochondria undergo profound remodeling in the early weeks of life, evidenced by strong growth, alterations in dynamics, and biogenic signaling of mitochondria [27, 28]. Perturbations during this susceptible phase of mitochondrial development can result in severe cardiac defects and pump failure [28]. Thus, it seems very likely that a profound and persistent intervention such as complete deletion of SIRT5, a protein regulating multiple energy metabolic pathways within mitochondria, may have deleterious effects if hearts are energetically challenged in later life. Conversely, the heart may cope much better with the onset of SIRT5 deletion in an adult stage. Since we observed a marked phenotype in mice with cardiomyocyte‐selective SIRT5 deletion (in contrast to Hershberger et al. [10]), multitissue effects are unlikely to explain why postnatal deletion of SIRT5 did not cause any detrimental cardiac phenotype [10]. Conduction of functional and molecular studies during the prenatal and early postnatal phase using our transgenic model may help to further characterize the actual impact or to identify novel targets of SIRT5.

In contrast to *cSirt5* ^*−/−*^ mice, cardiomyocyte‐restricted overexpression of SIRT5 showed no abnormalities in cardiac function or structure following TAC in our study. Guo et al. demonstrated that overexpression of SIRT5 can even be protective against pressure overload induced LV dilation, cardiomyocyte hypertrophy, and systolic dysfunction [12]. The differences between the two studies may be related to the use of profoundly older (4 to 8 months) mice by Guo et al. or may be attributed to the whole‐body SIRT5 overexpression model employed in that study. In addition, protein succinylation was not reduced in our study (for unknown reasons), but in the study of Guo and colleagues [12], implying that the magnitude of baseline succinylation may be relevant to achieve beneficial effects of SIRT5 overexpression. Loss of SIRT5 enzymatic activity in our animal model is unlikely, given that our *cSirt5* ^*−/−*^ mice were generated using floxed SIRT5 transgenic mice also used in the study by Guo et al. Nonetheless, both studies highlight the important finding that enhanced SIRT5 activation is not detrimental and may bear therapeutic potential in a setting of impaired SIRT5 function to be explored in future studies.

Using unbiased metabolomics, we detected increased levels of myocardial inosine and hypoxanthine and decreased levels of ATP precursors, ADP and AMP, in *cSirt5* ^*−/−*^ mice. A progressive decline in total adenine nucleotides has been previously observed in heart failure models. In the rapid right ventricular pacing model, a decrease in ATP levels correlated with decreased AMP and ADP levels, and an inhibition of de novo purine synthesis has been proposed to underlie adenine nucleotide depletion [16]. In spontaneously hypertensive rats, only a subtle impairment of ADP supplies was sufficient to limit oxidative ATP regeneration [29]. In addition, it has been hypothesized that depletion of intracellular AMP and ADP may impair activation of AMP‐activated protein kinase (AMPK) [30, 31] and phosphofructokinase (PFK) [32], thereby impairing both glycolytic and oxidative energy metabolism in the heart. Thus, synergistic effects of adenine nucleotide depletion and additionally increased energy demands due to chronic pressure overload may have impaired overall ATP turnover to an extent that facilitates aggravation of cardiac dysfunction in *cSirt5* ^*−/−*^ mice following pressure overload.

In contrast to pacing‐induced HF [16], we observed an accumulation of inosine and hypoxanthine in *cSirt5* ^*−/−*^ hearts, associated with increased mRNA levels of NT5E and PNP. NT5E hydrolyzes AMP to form adenosine [33] and downstream metabolites such as inosine and hypoxanthine, and PNP converts inosine to hypoxanthine, suggesting that increased NT5E and PNP may contribute to purine metabolite accumulation in *cSirt5* ^*−/−*^ hearts. Interestingly, increased myocardial NT5E expression has also been observed in HF patients [34, 35]. While the functional consequence of NT5E upregulation in the heart remains to be elucidated, mechanistic insights from loss‐of‐function models suggest both protective and maladaptive effects. Deficiency of NT5E has been shown to exacerbate pressure overload‐induced LV hypertrophy and dysfunction [36], particularly upon enhanced production of proinflammatory cytokines by T cells in the injured myocardium [37], whereas inhibition of NT5E attenuated ischemia reperfusion injury in rabbits attributed to maintenance of ATP precursor availability [38]. In addition to increased NT5E levels in *cSirt5* ^*−/−*^ hearts, decreased expression of ADK could also increase inosine levels but also impair adenosine conversion to AMP, thereby causing or amplifying adenosine nucleotide depletion. Collectively, we hypothesize that SIRT5 deficiency drives a transcriptional dysregulation of enzymes of purine metabolism at the interface of adenosine nucleotide salvage and purine degradation, thereby impairing ATP turnover and aggravating cardiac deterioration (Figure 4F). Previous reports showing accumulation of purine metabolites (e.g., adenosine, adenine, xanthine) and decreased expression of key enzymes of purine *de novo* synthesis (phosphoribosyl pyrophosphate synthetase, PRPS1, and phosphoribosyl pyrophosphate amidotransferase, PPAT) in cerebral cortex of mice lacking SIRT5 may support a direct regulation of enzymes of purine metabolism by SIRT5 deficiency [39].

Using LV samples of HFpatients and of healthy, age‐ and sex‐matched non‐failing control patients, we found decreased levels of SIRT5 in failing human hearts. Based on the fact that SIRT5 is a regulatory enzyme that influences many downstream metabolic pathways and the necessity of a fine‐tuned regulation of metabolism in an organ with continuously high but also changing energy demands, even mild chronic changes of SIRT5 expression may have a significant impact on the pathophysiology of human cardiac disease over time. While the expression of NT5E was not increased in ours but other studies [34, 35], we did find decreased myocardial expression of ADK, which correlated with decreased myocardial expression of SIRT5. Interestingly, cardiomyocyte‐specific *Adk* ^*−/−*^ mice display aggravation of cardiac hypertrophy and development of symptomatic dilative cardiomyopathy in response to chronic pressure overload [40]. Others showed that purine degradation products (ADP, AMP, inosine, hypoxanthine, and xanthine) can be released by the myocardium [41] and that systemic changes in purine metabolites (including inosine and hypoxanthine) have been observed in patients with dilated [18] or hypertrophic [42, 43, 44] cardiomyopathy and HF [45]. Collectively, our animal and human data suggest that decreased levels of SIRT5 in failing human hearts may drive downregulation of ADK, potentially aggravating myocardial energy depletion and promoting disease progression in HF.

## 5 Conclusions

Here we show that loss of SIRT5 in cardiomyocytes aggravates myocardial remodeling and dysfunction upon chronic pressure overload, possibly by ATP precursor depletion due to transcriptional dysregulation of cardiac purine metabolism. Murine and human data suggest that reduced levels of SIRT5 in failing human hearts may drive downregulation of ADK, thereby aggravating myocardial energy depletion and progression of HF. Since SIRT5 overexpression is not harmful and may even protect from HF progression [12], maintaining or restoring normal SIRT5 levels remains a promising therapeutic target for HF patients.

## Conflicts of Interest

The authors declare no conflicts of interest.

## Supporting information


**Data S1:** apha70120‐sup‐0001‐DataS1.zip.

## Data Availability

The data that support the findings of this study are available from the corresponding author upon reasonable request.
