# Supplementary material for: Cardiomyocyte‐Specific Deletion of Sirtuin 5 Accelerates the Development of Heart Failure Upon Dysregulating Purine Metabolism
Source: Acta Physiol (Oxf). 2025 Oct 17;241(11):e70120. doi: 10.1111/apha.70120 (PMC12533344; doi:10.1111/apha.70120)

Supplemental Figure 1

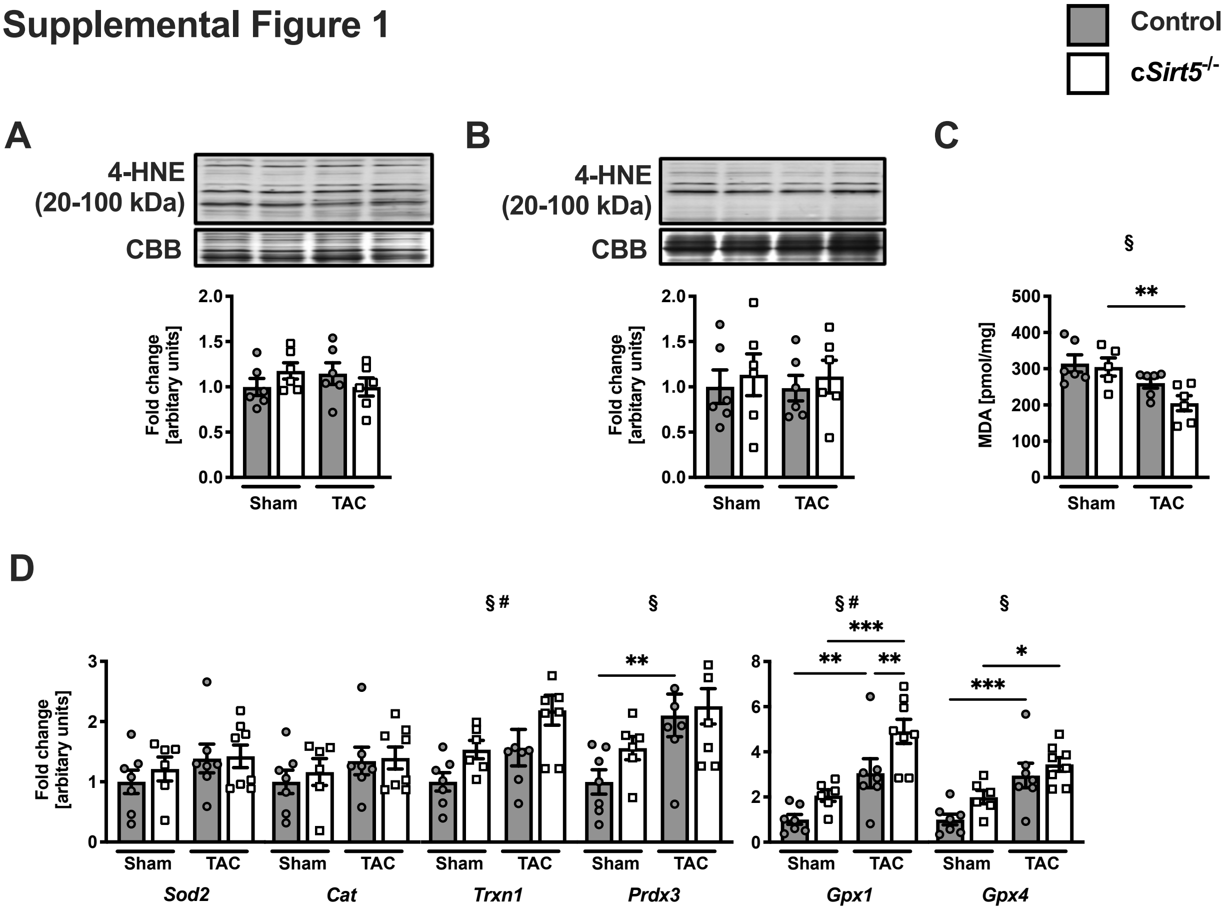

Supplemental Figure 2

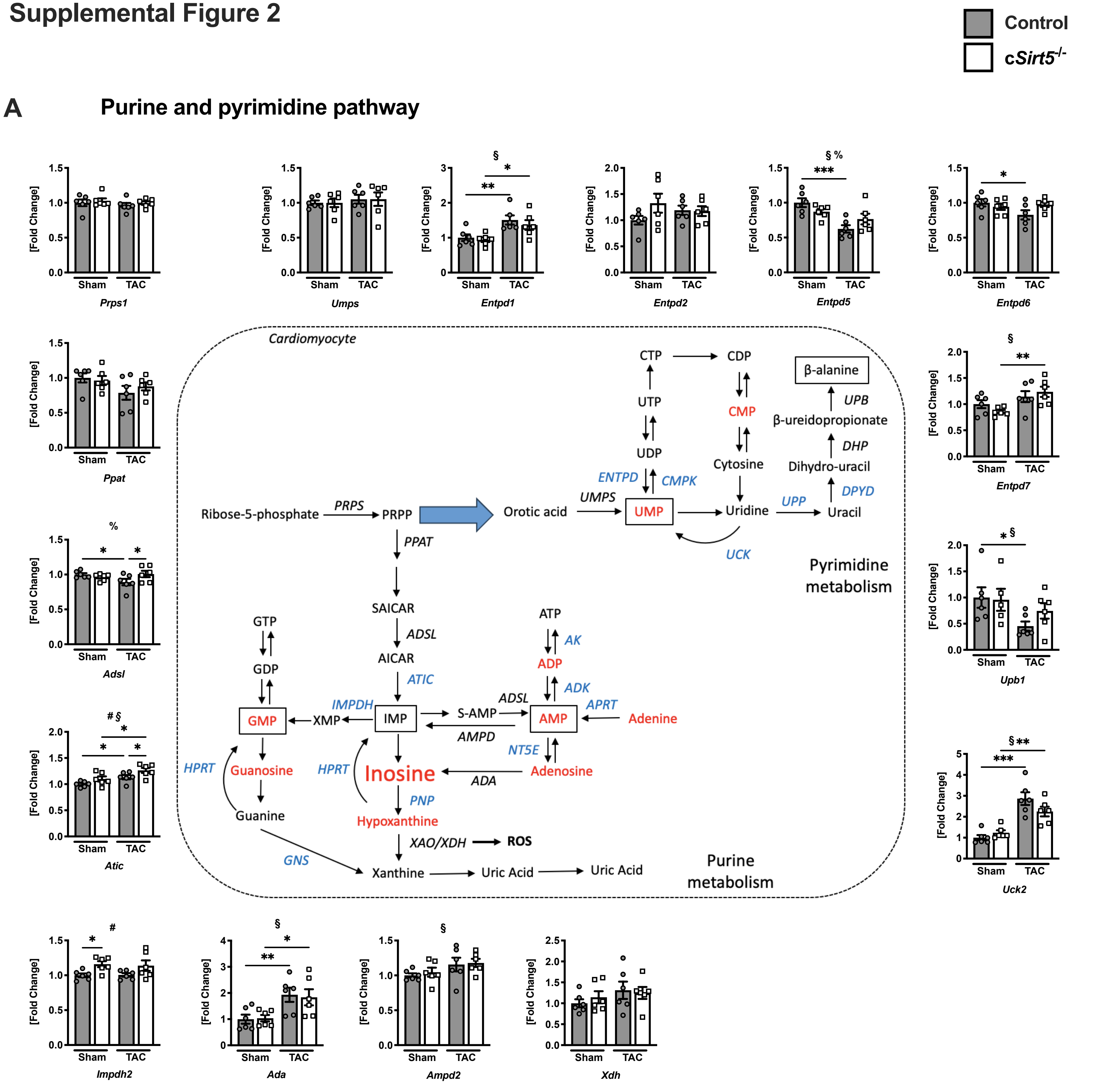

Supplemental Figure 3

Supplemental Full Western Blot Images: **Figure 1A**

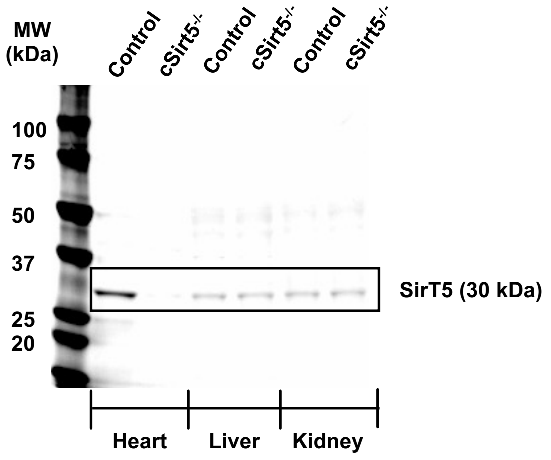

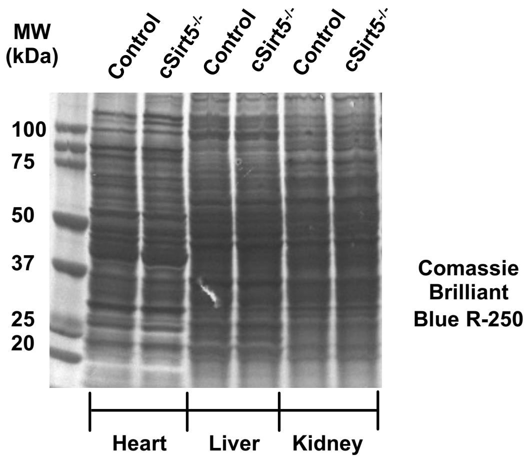

Supplemental Full Western Blot Images: **Figure 2A**

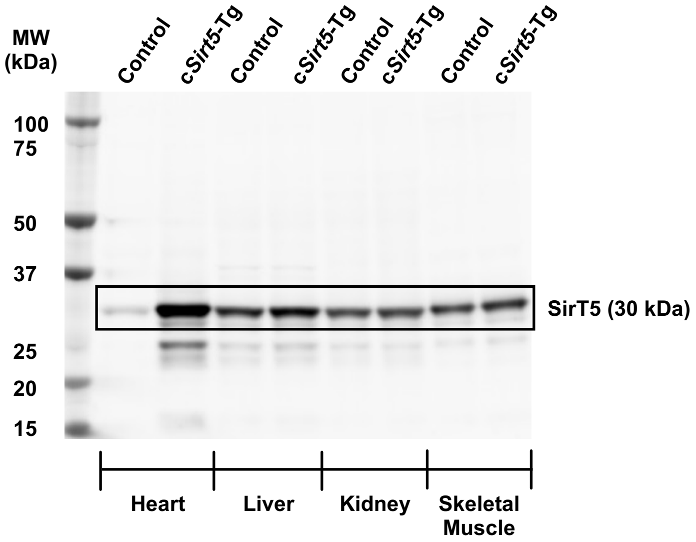

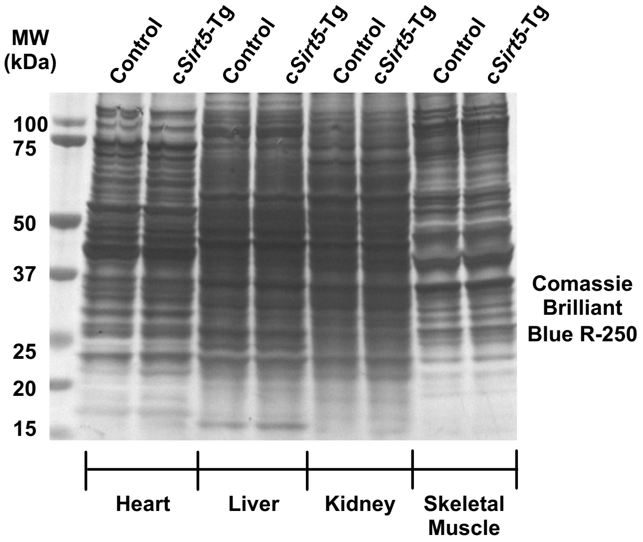

Supplemental Full Western Blot Images: **Figure 2B**

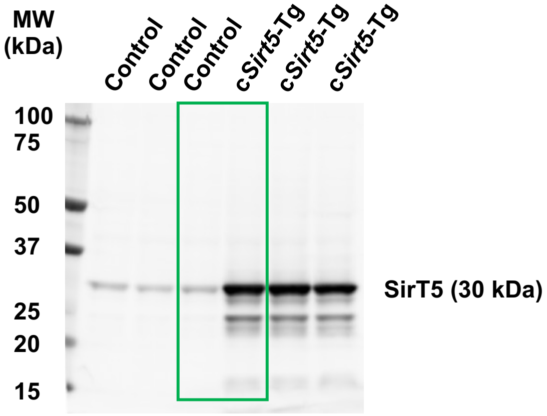

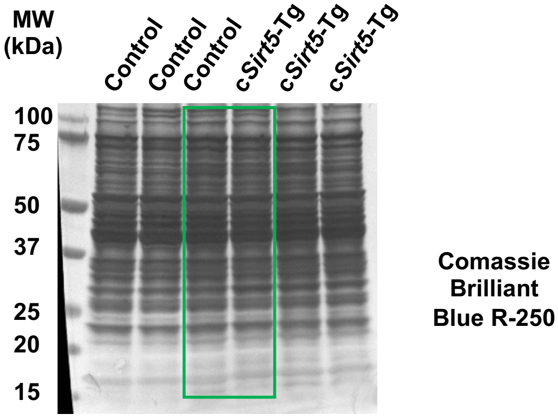

Supplemental Figure 3 con't

Supplemental Full Western Blot Images: **Figure 1C and 1D / Figure 2C and 2D**

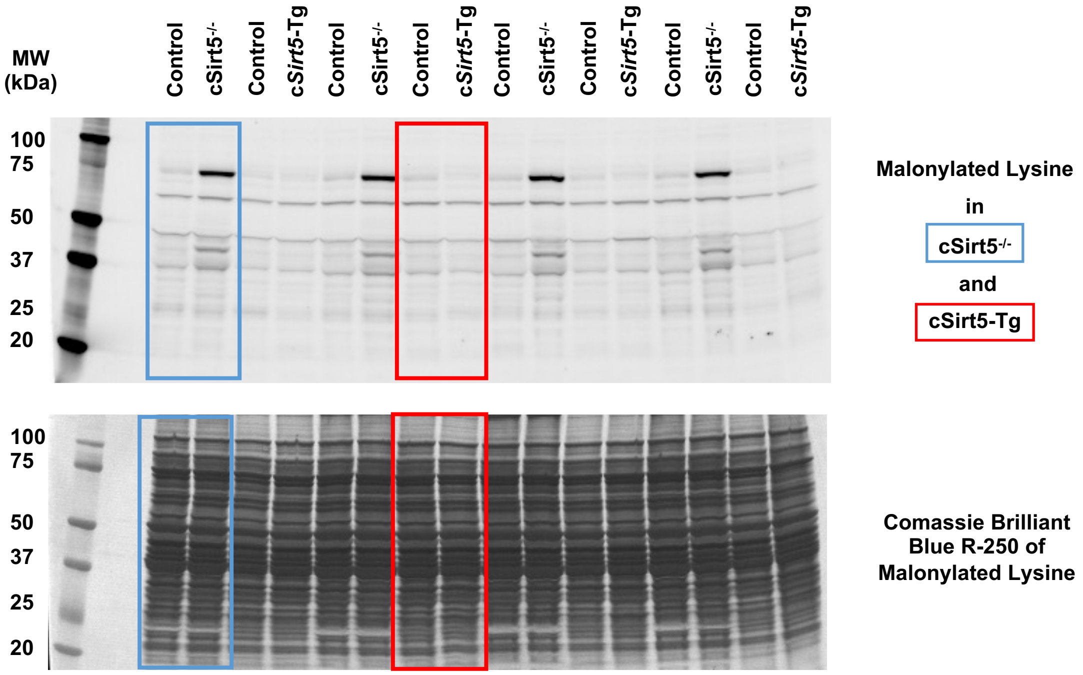

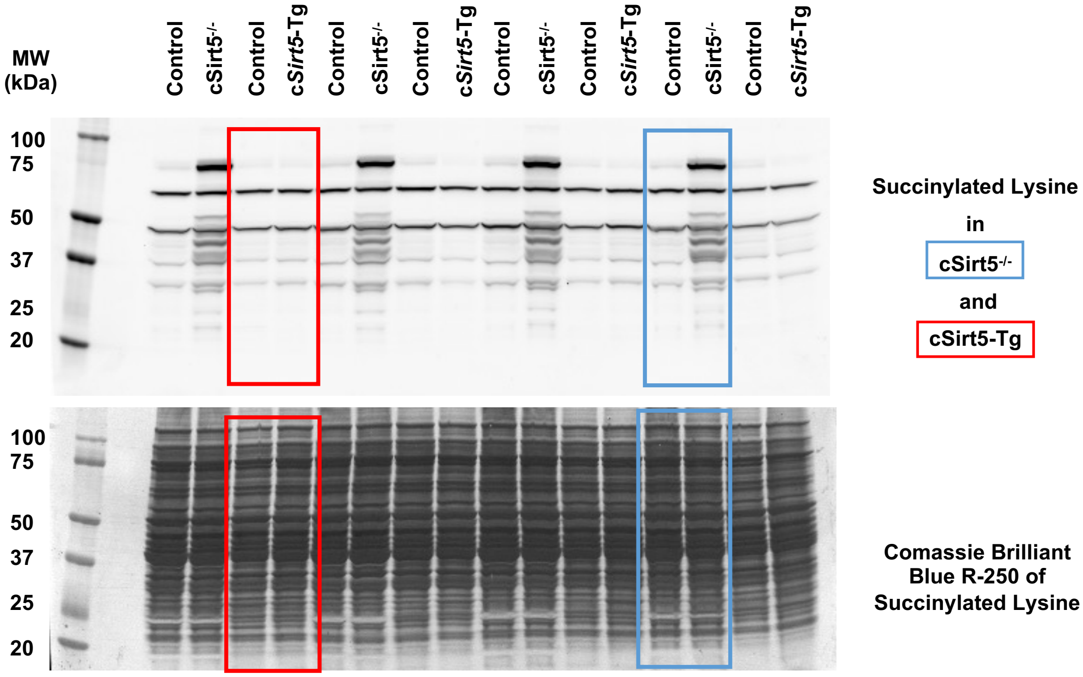

Supplemental Figure 3 con't

Supplemental Full Western Blot Images: **Figure 5B, 5D and 5F**

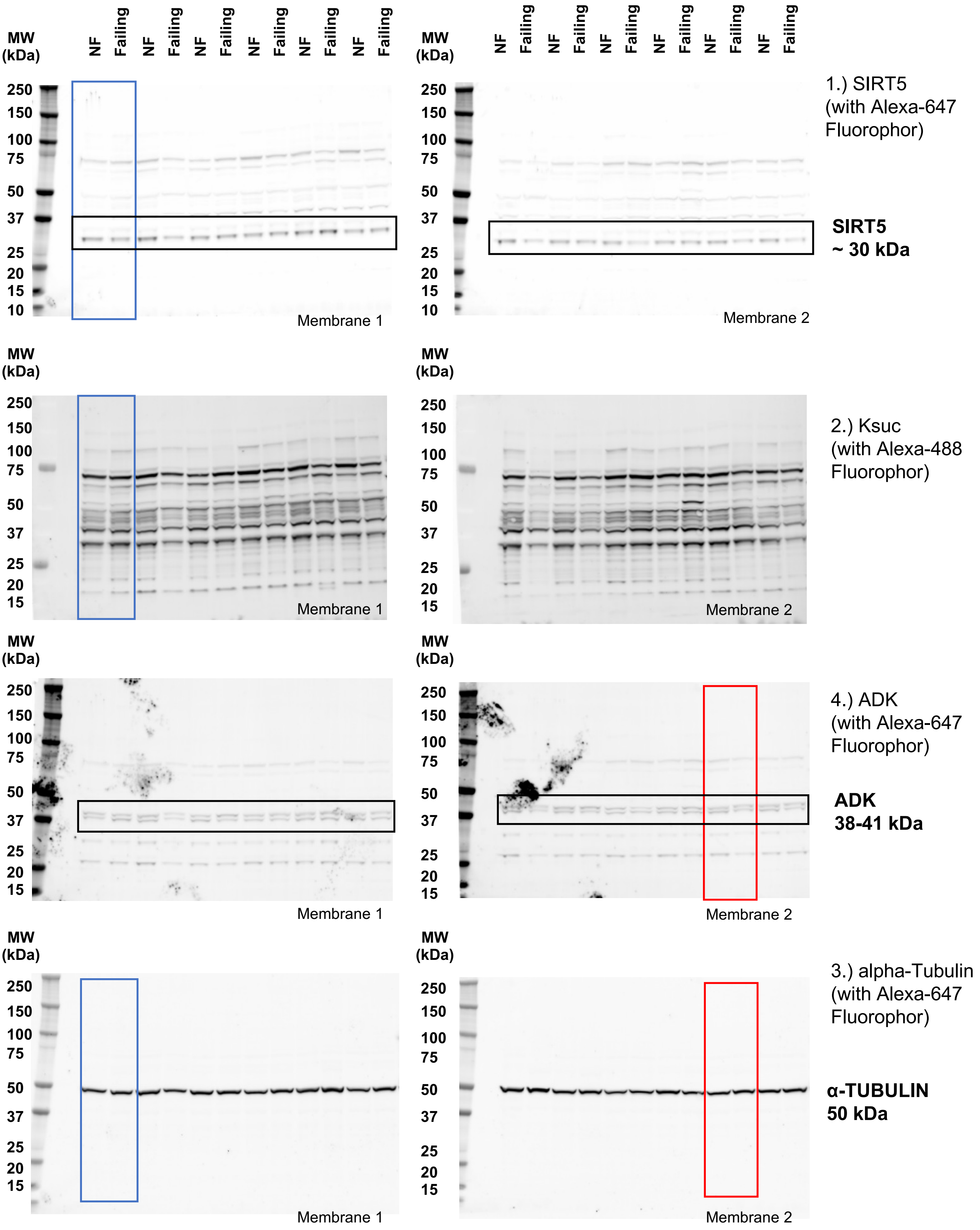

Supplemental Figure 3 con't

Supplemental Full Western Blot Images: **Figure 1B and 4E**

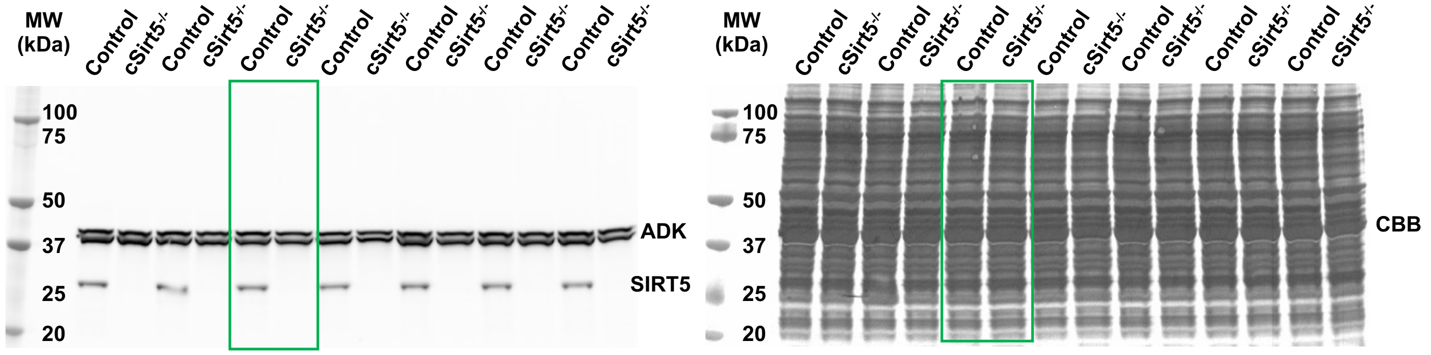

Supplemental Full Western Blot Images: **Supplemental Figure 2A**

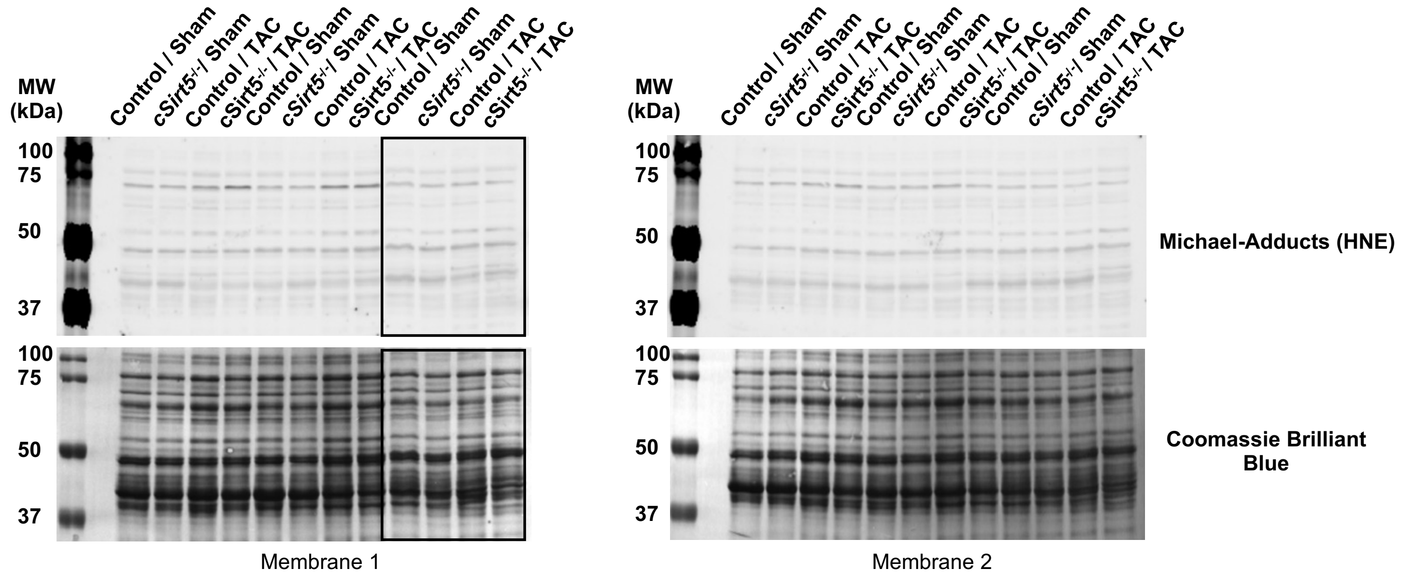

Supplemental Full Western Blot Images: **Supplemental Figure 2B**

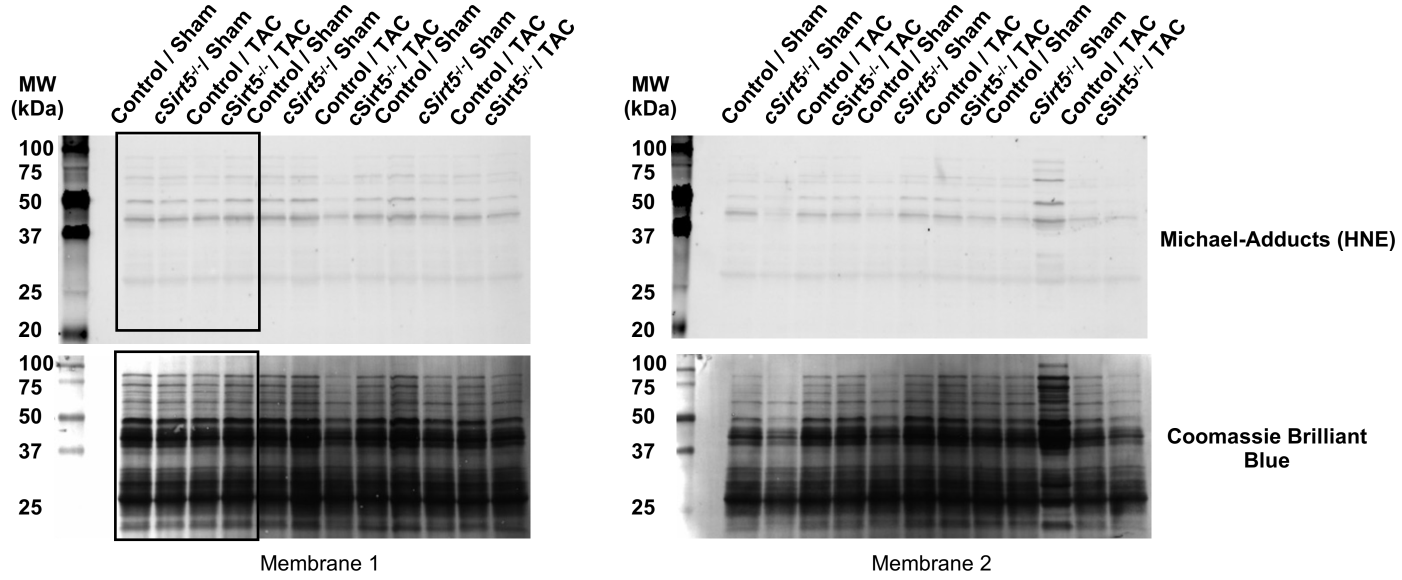

Supplement: Supplementary file 1 — Data S1: apha70120‐sup‐0001‐DataS1.zip. [file APHA-241-e70120-s001.zip › apha70120-sup-0001-DataS1/Suppl Figures 1-3 - SIRT5 HF Acta Phys.pdf]
