# Supplementary material for: Cardiomyocyte‐Specific Deletion of Sirtuin 5 Accelerates the Development of Heart Failure Upon Dysregulating Purine Metabolism
Source: Acta Physiol (Oxf). 2025 Oct 17;241(11):e70120. doi: 10.1111/apha.70120 (PMC12533344; doi:10.1111/apha.70120)
Supplement: Supplementary file 1 — Data S1: apha70120‐sup‐0001‐DataS1.zip. [file APHA-241-e70120-s001.zip › apha70120-sup-0001-DataS1/Supplemental SIRT5 HF revised - Acta Phys.docx]

**Supplemental Materials and Methods**

**Transverse aortic constriction (TAC) -** Minimally-invasive transverse aortic constriction was performed as described previously.^1^ Mice were anesthetized with a single intraperitoneal injection of ketamine (100 mg/kg) and xylazine (6 mg/kg). Following fixation in supine position, a craniocaudal skin incision of approx. 5–10 mm in length was made. The salivary glands were retracted, and a partial thoracotomy was performed by longitudinal incision of the cranial sternum (2–3 mm). The microsurgical clip (internal diameter 0.305 mm) was applied on the transverse aorta between the left carotid artery and the brachiocephalic trunk using a micro clip applicator, adjusted to an internal diameter of 0.23 mm (outer diameter of a 32G needle) for consistency. Successful constriction was confirmed by a visible increase in perfusion of the right carotid artery immediately after clip application. The thorax was closed using suture, and mice were allowed to recover on a warming pad until they were fully awake. The sham procedure was performed in identical fashion except for clip application. After 8 and 12 weeks, respectively, c*Sirt5*-Tg and c*Sirt5*^-/-^ mice and their controls were anesthetized using 0.3 mg/g body weight thiopenthal i.p. and hearts were excised for further experiments.

**Transthoracic echocardiography -** Transthoracic echocardiography was performed before start of treatment and up to 8 and 12 weeks of TAC on c*Sirt5*-Tg and c*Sirt5*^-/-^ mice, respectively, and their respective controls as described previously.^1^ Mice were anesthetized using isoflurane inhalation (3 % for induction and 2 % for maintenance of anesthesia). M-Mode images and 2D parasternal short axis images were taken using a Vivid 7 Dimension (GE Healthcare, Munich, Germany) micro-imaging system equipped with an i13L transducer (14 MHz). Ejection fraction and LV geometry were quantified as described previously.^1^

**Isolated working heart perfusion -** Hearts were excised and examined by isolated working mouse heart perfusion, as described previously.^2^ In brief, hearts were perfused using Krebs-Henseleit Buffer (KHB) containing (in mM) 128 NaCl, 5 KCl, 1 KH_2_PO_4_, 1.3 MgSO_4_, 15 NaHCO_3_, 2.5 CaCl_2_, 0.4 palmitate (bound to 3 % BSA), and 5 glucose at 37C, with 50 mmHg afterload and 15 mmHg preload. Aortic pressure changes were measured using a pressure catheter placed inside the aortic cannula (Millar Micro-Tip, Millar Instruments). Cardiac output and cardiac power were quantified to evaluate contractile function. Myocardial oxygen consumption (MVO_2_) was measured as difference of percent oxygen concentration in pre- and post-cardial buffer samples using a fiber-optic oxygen sensor (Ocean Optics). Cardiac efficiency was calculated as ratio of hydraulic work to MVO_2_.

**RNA extraction and quantitative RT-PCR -** Total RNA was extracted from hearts of c*Sirt5*^-/-^ and c*Sirt5*-Tg mice following Sham and TAC using TRIzol reagent (Qiagen), followed by extraction using chloroform. RNA was purified by using the RNeasy Kit (Qiagen) according manufacturing protocol with some modifications. Reverse Transcription was performed by using SuperScript III Reverse Transcriptase Kit (Invitrogen). iQ SYBR Green Supermix (BioRad) was used as probe, and amplification was monitored using the CFX96 Real-Time PCR system (BioRad). Data were normalized by expressing relative to levels of the invariant transcripts of the 60S acidic ribosomal protein P0 (Rplp0) in mice and ribosomal protein S16 (RPS16) in human, respectively and are presented as arbitrary units normalized to the expression levels of the Control Sham group. Primer sequences are presented in **Supplemental Table 1-2**.

**Western Blot Analysis -** Frozen tissue from mouse heart, liver, kidney and skeletal muscle were homogenized in buffer containing (in mM) 50 HEPES, 150 NaCl, 10 % glycerol, 1 % Triton X‑100, 1.5 MgCl_2_, 1 EGTA, 100 NaF, Complete Protease inhibitor cocktail (Roche Life Science) and Phosphatase inhibitor cocktails 2 and 3 (Sigma-Aldrich), pH 7.5 using Ultra-Turrax T10 homogenizer. Mitochondria were isolated from fresh excised hearts from c*Sirt5^-/-^* and Control mice following TAC and Sham by differential centrifugation using an isolation buffer containing (in mM) 250 sucrose, 5 Tris HCl and 2 EGTA, pH 7.4. Mitochondrial samples were additionally treated with Halt Protease and Phosphatase Inhibitor Cocktail (Thermo Fisher Scientific). Protein concentrations of the supernatant and mitochondria were determined using a BCA reagent (Thermo Fisher Scientific) with BSA as standard. Proteins were separated by SDS-PAGE, transferred onto PVDF membranes (BioRad) and incubated with primary antibodies anti-SIRT5 (8782, Cell Signaling Technology), anti-malonyl-lysine (PTM-901, PAN-Biotech) anti-succinyl-lysine (PTM-401, PAN-Biotech), anti‑4‑Hydroxynonenal (393204, Merck-Millipore), anti-ADK (93994, Cell Signaling Technology), anti-α-Tubulin (T9026, Merck Group) at 4°C overnight. Secondary antibodies anti‑Rabbit IgG (H+L) F(ab’)_2_ Alexa Fluor 647 Conjugate (4414, Cell Signaling Technology) or anti‑Mouse IgG (H+L) F(ab’)_2_ Alexa Fluor 488 Conjugate (4408, Cell Signaling Technology) were incubated for 1 hour at room temperature in the dark. Detection and quantification of fluorescent bands was performed using the Bio-Rad Western Blot Imager. Loading control was performed using staining with Coomassie Brilliant Blue R-250 or α-Tubulin. Uncropped images are presented in **Supplemental Figure 3**.

**Histology -** Excised hearts were snap frozen in methylbutane in liquid nitrogen and embedded in Tissue-Tek OCT cryo embedding compound. Cardiomyocyte size and the degree of fibrosis were determined in 12 µm sections using wheat germ agglutinin (WGA) staining (Thermo Fisher Scientific) and Masson’s trichrome staining (Sigma-Aldrich), respectively. 3-4 random sections were imaged blindly at 40X and 20X magnification for WGA and Masson’s trichrome, respectively. Cardiomyocyte size was determined by measuring 30 cardiomyocytes using AxioVision Rel 4.6 (Carl Zeiss NTS GmbH) and Masson’s Trichrome. using ImageJ software.

**Thiobarbituric acid reactive substances (TBARS) -** Frozen heart tissue was homogenized in ice-cold PBS containing 120 mM KCL (pH = 7.4) using Ultra-Turrax T10 homogenizer and centrifuged at 1,600 x g for 10 min at 4°C. 100 µl of the supernatant was incubated with 200 µl 100 mM HCl, 30 µl phosphotungstic acid (10 % w/v) and 100 µl thiobarbituric acid (0.7 % M) for 5 min at room temperature, followed by another incubation for 60 min at 95 °C. To stop the reaction, samples were put on ice and 500 µl 1‑butanol was added to extract TBARS. Following centrifugation for 10 min at 1,000 x g at 4°C, fluorescence of the butanolic layer was measured at 515 nm (excitation) and 553 nm (emission). Tetrametoxipropane was used as a standard to calculate TBARS content.

**References**

**1.** Koentges C, Pepin ME, Musse C, et al. Gene expression analysis to identify mechanisms underlying heart failure susceptibility in mice and humans. *Basic Res Cardiol.* 2018;113:8.

**2.** Koentges C, Pfeil K, Schnick T, et al. SIRT3 deficiency impairs mitochondrial and contractile function in the heart. *Basic Res Cardiol.* 2015;110:36.

**Supplemental Figure legends**

**Supplemental Figure 1: Oxidative stress and antioxidant expression in c*Sirt5^-/-^* mice following TAC. A)** Total myocardial and **B)** mitochondrial 4-HNE levels and **C)** myocardial TBARS assay in control and *cSirt5^-/-^* mice 12 weeks following Sham or TAC surgery (n=6). **D)** Myocardial expression of antioxidant genes in control and *cSirt5^-/-^* mice 12 weeks following Sham or TAC surgery (n=6-8). 4-HNE, 4-hydroxynonenal; c*Sirt5*^-/-^, cardiomyocyte-specific deletion of *Sirt5*; CAT, catalase; GPX, glutathione peroxidase; PRDX, peroxidase; Sod, superoxide dismutase; TRXN, thioredoxin; Sirt5, sirtuin 5; TAC, transverse aortic constriction. 2-Way ANOVA: §, effect of TAC; #, effect of c*Sirt5*^-/-^; % effect of interaction. *p<0.05, **p<0.01, ***p<0.001 using Fisher’s LSD test.

**Supplemental Figure 2:** **Purine and pyrimidine gene expression in c*Sirt5*^-/-^ hearts following TAC.** **A)** Expression of genes encoding for proteins involved in purine and pyrimidine metabolism in control and c*Sirt5*^-/-^ mice at 12 weeks following Sham or TAC surgery (n=6). Altered metabolites in c*Sirt5*^-/-^ hearts were indicated in red; altered genes in c*Sirt5*^-/-^ hearts were indicated in blue. ADA, adenosine deaminase; ADP, adenosine diphosphate; ADSL, adenylosuccinate lyase; AMP, adenosine monophosphate; AMDP, AMP deaminase 3; APRT, adenine phosphoribosyl transferase; APRT, adenine phosphoribosyl transferase; ATIC, 5-aminoimidazole-4-carboxamide ribonucleotide formyltransferase/IMP cyclohydrolase; ATP, adenosine triphosphate; CDP, cytidine diphosphate; CMP, cytidine monophosphate; CTP, cytidine triphosphate; DHP, dihydropyrimidinase; ENTPD, ectonucleoside triphosphate diphosphohydrolase; FAD, flavin adenine dinucleotide; GCLM, glutamate-cysteine ligase modifier subunit; GDP, guanosine diphosphate; GMP, guanosine monophosphate; GMPS, GMP synthetase; GTP, guanosine triphosphate; IMP, inosine monophosphate; IMPDH, IMP dehydrogenase; PPAT, phosphoribosyl pyrophosphate amidotransferase; PRPS, phosphoribosyl pyrophosphate synthetase; SIRT5, sirtuin 5; TAC, transverse aortic constriction; UCK, uridine-cytidine kinase; UDP, uridine diphosphate; UMP, uridine monophosphate; UMPS, UMP synthetase; UPB, ureidopropionase, beta; UTP, uridine triphosphate; XDH, xanthine dehydrogenase; XO, xanthine oxidase. 2-Way ANOVA: §, effect of TAC; #, effect of c*Sirt5*-Tg; % effect of interaction. *p<0.05, **p<0.01, ***p<0.001 using Fisher’s LSD test.

**Supplemental Tables**

**Supplemental Table 1:** Heart weight of control and cSirt5-/- mice 12 weeks following Sham or TAC surgery.

|  | Control | | | *Sirt5*^-/-^ | | | Control | | | *Sirt5*^-/-^ | | | 2-Way |
| --- | --- | --- | --- | --- | --- | --- | --- | --- | --- | --- | --- | --- | --- |
|  | Sham | | | Sham | | | TAC | | | TAC | | | ANOVA |
| BW (g) | 28.3 | ± | 0.3 | 27.4 | ± | 0.5 | 29.5 | ± | 0.5 | 28.3 | ± | 0.4 |  |
| TL (mm) | 17.7 | ± | 0.1 | 17.7 | ± | 0.1 | 17.9 | ± | 0.1 | 17.7 | ± | 0.1 |  |
| HW (mg) | 118.0 | ± | 2.6 | 108.4 | ± | 2.8 | 190.1 | ± | 10.4* | 172.6 | ± | 10.8* | § |
| HW/BW (mg/g) | 4.17 | ± | 0.09 | 3.96 | ± | 0.06 | 6.46 | ± | 0.34* | 6.15 | ± | 0.44* | § |
| HW/TL (mg/mm) | 6.67 | ± | 0.15 | 6.12 | ± | 0.13 | 10.64 | ± | 0.57* | 9.75 | ± | 0.60* | § |

BW, body weight; HW, heart weight; TAC, transverse aortic constriction; TL, tibia length. Mean ± SEM; n=14 Control Sham; n=10 c*Sirt5*^-/-^ Sham; n=15 Control TAC; n=11 c*Sirt5*^-/-^ TAC; 2-Way ANOVA: §, effect of TAC; #, effect of c*Sirt5*^-/-^; % effect of interaction. *p<0.05 versus respective Sham, †p<0.05 versus control TAC using post-hoc test.

**Supplemental Table 2:** Heart weight of control and c*Sirt5*-Tg mice 8 weeks following Sham or TAC surgery.

|  | Control | | | c*Sirt5-*Tg | | | Control | | | c*Sirt5-*Tg | | | 2-Way |
| --- | --- | --- | --- | --- | --- | --- | --- | --- | --- | --- | --- | --- | --- |
|  | Sham | | | Sham | | | TAC | | | TAC | | | ANOVA |
| BW (g) | 26.4 | ± | 0.9 | 25.9 | ± | 1.0 | 27.6 | ± | 0.9 | 27.9 | ± | 1.1 |  |
| TL (mm) | 17.8 | ± | 0.2 | 17.8 | ± | 0.2 | 18.2 | ± | 0.2 | 18.3 | ± | 0.13* | § |
| HW (mg) | 128.5 | ± | 6.4 | 126.9 | ± | 6.9 | 191.8 | ± | 8.49* | 196.8 | ± | 10.3* | § |
| HW/BW (mg/g) | 4.85 | ± | 0.11 | 4.9 | ± | 0.19 | 6.93 | ± | 0.08* | 7.05 | ± | 0.27 | § |
| HW/TL (mg/mm) | 7.19 | ± | 0.31 | 7.14 | ± | 0.36 | 10.53 | ± | 0.37* | 10.73 | ± | 0.53* | § |

BW, body weight; HW, heart weight; TAC, transverse aortic constriction; TL, tibia length. Mean ± SEM; n=14 Control Sham; n=10 c*Sirt5*-Tg Sham; n=15 Control TAC; n=11 c*Sirt5*-Tg TAC; 2-Way ANOVA: §, effect of TAC; #, effect of cSirt5-Tg; % effect of interaction. *p<0.05 versus respective Sham, †p<0.05 versus control TAC using post-hoc test.

**Supplemental Table 3:** Sequences of forward and reverse primers used for reverse transcription quantitative polymerase chain reaction in mice.

| **Gene** | **Primer** | **Primer Sequence (5´-3´)** |
| --- | --- | --- |
| *Sod2* | Forward | ACAACTCAGGTCGCTCTTCA |
|  | Reverse | GAACCTTGGACTCCCACAGA |
| *Cat* | Forward | AGCGACCAGATGAAGCAGTG |
|  | Reverse | TCCGCTCTCTGTCAAAGTGTG |
| *Txn1* | Forward | GCCAAAATGGTGAAGCTGAT |
|  | Reverse | TGATCATTTTGCAAGGTCCA |
| *Prdx3* | Forward | ACGGAGTGCTGTTGGAAAGT |
|  | Reverse | TTGATCGTAGGGGACTCTGG |
| *Gpx1* | Forward | GTCCACCGTGTATGCCTTCT |
|  | Reverse | TCACCATTCACTTCGCACTT |
| *Gpx4* | Forward | ATGCCCGATATGCTGAGTGT |
|  | Reverse | CGGCAGGTCCTTCTCTATCA |
| *Rplp0* | Forward | AAGCGCGTCCTGGCATTGTCT |
|  | Reverse | CCGCAGGGGCAGCAGTGGT |

CAT, catalase; GPX, glutathione peroxidase; PRDX, thioredoxin-dependent peroxide reductase; RPLP0, ribosomal protein large P0; SOD, superoxide dismutase; TRXN, thioredoxin.

**Supplemental Table 4:** Sequences of forward and reverse primers used for reverse transcription quantitative polymerase chain reaction in human.

| **Gene** | **Primer** | **Primer Sequence (5´-3´)** |
| --- | --- | --- |
| *SIRT5* | Forward | TGCCATTCTGGAGGAGGTTG |
|  | Reverse | AAACATGGCTGCTGGGTACA |
| *ADA* | Forward | CGGGACATGGGCTTTACTGA |
|  | Reverse | GTCGAGAAGCTCCCTCTTTTCA |
| *IMPDH1* | Forward | GCATCCAGACCGTGGGACA |
|  | Reverse | AGGGAGCCCATCATCACTGT |
| *ADARB1* | Forward | GACCCTGTCTGTCAACACGG |
|  | Reverse | CCCACGTAAAAGGGAGGCTC |
| *ENTPD5* | Forward | GCAACAGCAGGACTACGCTTA |
|  | Reverse | TGCTAACACTGCCCTTTGGTA |
| *UPP1* | Forward | CATCCGCATTGGCACTTCTG |
|  | Reverse | GCAGAACACAGCAACAGCTC |
| *DPYD* | Forward | ACAGCCACCAACACTGTCTC |
|  | Reverse | ATGGGAAATCCAGGCAGAGC |
| *NT5E* | Forward | AGGTTCCACCCTGAAGAAGG |
|  | Reverse | CACTCGACACTTGGTGCAAA |
| *ADK* | Forward | TTAAAGGTGGCTCACCATGC |
|  | Reverse | AAGTGGCAGCTTCTGTCTCA |
| *RPS16* | Forward | CAGCGCTCCTGTACCCTTTA |
|  | Reverse | AGCAGGGTCCGGTCATACT |

ADARB, adenosine deaminase RNA specific B; ADA, adenosine deaminase; ADK, adenosine kinase; DPYD, dihydropyrimidine dehydrogenase; ENTPD, ectonucleoside triphosphate diphosphohydrolase; IMPDH1, inosine monophosphate dehydrogenase; NT5E, 5' nucleotidase, ecto; RPS16, ribosomal protein S16; SIRT5, sirtuin 5; UPP, uridine phosphorylase.

**Supplemental Table 5:** Patient characteristics of non-failing and HF patients.

| Characteristics | Non-failing | | | Failing | | |
| --- | --- | --- | --- | --- | --- | --- |
| N | 19 | | | 15 | | |
| Age [y] | 60.4 | ± | 9.5 | 50.7 | ± | 10.6 |
| Female [N] | 5 | | | 5 | | |
| BMI [kg/m2] | 26.2 | ± | 3.9 | 25.6 | ± | 4.2 |
| Type 2 Diabetes [N] | 1 | | | 3 | | |
| Hypertension [N] | 10 | | | 6 | | |
| EF [%] | 63.8 | ± | 5.8 | 27.2 | ± | 11.2 |
